# Missense variants in *TUBA4A* cause myo-tubulinopathies

**DOI:** 10.1093/brain/awag059

**Published:** 2026-10-05

**Authors:** Mridul Johari, Chiara Folland, Yoshihiko Saito, Machteld M. Oud, Jevin M. Parmar, Ana Töpf, Sergei Kurbatov, Maria Ampleeva, Ekaterina Y. Zakharova, Irina A. Chekmareva, Ksenia S. Shirokova, Dmitrii Atiakshin, Ronald van Beek, Thatjana Gardeitchik, Erik-Jan Kamsteeg, Evita Medici, Laura Donker Kaat, Joe Nemoto, Hirofumi Komaki, Takashi Okabe, Yasuhiro Kimoto, Takeshi Tokito, Masaki Nakanowatari, Yasushi Oya, Christine C. Bruels, Seth A. Stafki, Elicia A. Estrella, Hannah R. Littel, Louis M. Kunkel, Peter B. Kang, Ikeoluwa Osei-Owusu, Lynn Pais, Melanie O’Leary, Christina Austin-Tse, Anne O’Donnell-Luria, Brian Mangilog, Casie A. Genetti, Zaheer M. Valivullah, Francesca Clementina Radio, Adele D’Amico, Andrea Ciolfi, Marco Tartaglia, Aurélien Perrin, Charles Van Goethem, Guilhem Sole, Marie-Laure Martin-Négrier, Mireille Cossée, Vedrana Milic Rasic, Gordana Kovacevic, Ana Kosac, Cristiane A. M. Moreno, Clara Gontijo Camelo, Edmar Zanoteli, Clair Habib, Michael C. Fahey, Alan H. Beggs, Nanna Scharff Poulsen, John Vissing, Volker Straub, Marco Savarese, Giorgio Tasca, Nicol C. Voermans, Nigel G. Laing, Bjarne Udd, Ichizo Nishino, Gianina Ravenscroft

**Affiliations:** 1 Harry Perkins Institute of Medical Research, Centre for Medical Research, University of Western Australia, Nedlands, WA, 6009, Australia; 2 Department of Neuromuscular Research, National Institute of Neuroscience, National Center of Neurology and Psychiatry, Kodaira, Tokyo, 187-8551, Japan; 3 Department of Human Genetics, Donders Institute for Brain, Cognition and Behaviour, Radboud University Medical Center, Nijmegen, 6525, The Netherlands; 4 John Walton Muscular Dystrophy Research Centre, NIHR Newcastle Biomedical Research Centre, Newcastle University and Newcastle Hospitals NHS Foundation Trust, Newcastle upon Tyne, NE4 5PL, UK; 5 Research Institute of Experimental Biology and Medicine, Voronezh State Medical University named after N.N. Burdenko, Voronezh, 394036, Russian Federation; 6 Independent Clinical Bioinformatics Laboratory, Moscow, Russia; 7 Research Centre for Medical Genetics, Moscow, 115522, Russia; 8 Federal State Budgetary Institution “National Medical Research Center of Surgery named after A. Vishnevsky”, Ministry of Health of the Russian Federation, Moscow, 117997, Russia; 9 RUDN University, Moscow, 117198, Russian Federation; 10 Department of Human Genetics, Radboud University Medical Center, Nijmegen 6525 GA, The Netherlands; 11 Department of Human Genetics, Radboudumc, Nijmegen, 6525, The Netherlands; 12 Department of Neurology, Erasmus University Medical Center Rotterdam, 3015, The Netherlands; 13 Department of Clinical Genetics, Erasmus University Medical Center Rotterdam, 3015, The Netherlands; 14 Department of Neurology, Yamaguchi University Graduate School of Medicine, Ube, Yamaguchi, 755-8505, Japan; 15 Translational Medical Center, National Center of Neurology and Psychiatry, Kodaira, Tokyo, 187-8551, Japan; 16 Okabe Children’s Clinic, Toyama, 939-8211, Japan; 17 Division of Pediatrics, Faculty of Medicine, University of Miyazaki, Miyazaki, 889-1692, Japan; 18 Tokito Clinic Rheumatology and Orthopedic Surgery, Shimonoseki, Yamaguchi, 752-0976, Japan; 19 Department of Neurology, Ushioda General Hospital, Yokohama, Kanagawa, 230-0001, Japan; 20 Department of Neurology, National Center Hospital, National Center of Neurology and Psychiatry, Kodaira, Tokyo, 187-8551, Japan; 21 Greg Marzolf Jr. Muscular Dystrophy Center, Department of Neurology, University of Minnesota Medical School, Minneapolis, MN 55455, USA; 22 Division of Genetics & Genomics, The Manton Center for Orphan Disease Research, Boston Children’s Hospital and Harvard Medical School, Boston, MA 02115, USA; 23 Broad Center for Mendelian Genomics, Program in Medical and Population Genetics, Broad Institute of MIT and Harvard, Cambridge, MA 02142, USA; 24 Molecular Genetics and Functional Genomics, Bambino Gesù Children’s Hospital, IRCCS, 00146, Rome, Italy; 25 Neuromuscular and neurodegenerative disorders, Bambino Gesù Children’s Hospital, IRCCS, 00146, Rome, Italy; 26 Laboratoire de Génétique Moléculaire, Centre Hospitalier Universitaire de Montpellier, 34093 Montpellier, France; 27 PhyMedExp, Univ Montpellier, CNRS, INSERM, Montpellier, 34295, France; 28 Neurology and Neuromuscular Diseases Department, Neuromuscular Reference Centre AOC, FILNEMUS, EURO-NMD, Pellegrin Hospital, Bordeaux University Hospitals, Bordeaux, 33000, France.; 29 Pathology Department, University Hospital of Bordeaux, France. Univ. Bordeaux, CNRS UMR5293, Bordeaux, 33076, France; 30 Clinic for Neurology and Psychiatry for Children and Youth, Faculty of Medicine, University of Belgrade, Belgrade, 11000, Serbia; 31 Mother and Child Health Care Institute, Faculty of Medicine, University of Belgrade, Belgrade, 11000, Serbia; 32 Department of Neurology, Faculdade de Medicina da Universidade de São Paulo (FMUSP), São Paulo, 05403-000, Brazil; 33 Clinical Genetics, Austin Health, Melbourne, VIC, 3084, Australia; 34 Department of Paediatrics Monash University, Melbourne, VIC, 3168, Australia; 35 Copenhagen Neuromuscular Center, Rigshospitalet, University of Copenhagen, Copenhagen, 2100, Denmark; 36 Folkhälsan Research Center, Helsinki, 00290, Finland; 37 Department of Neurology, Donders Institute for Brain, Cognition and Behaviour, Amalia Children’s Hospital, Radboud University Medical Centre, Nijmegen, 6525, The Netherlands

**Keywords:** tubulinopathy, tubulin, autophagy, genotype-phenotype correlation, protein aggregate myopathy

## Abstract

Tubulinopathies encompass a spectrum of disorders resulting from variants in genes encoding α- and β-tubulins, the key components of microtubules. While previous studies have linked *de novo* or dominantly inherited *TUBA4A* missense variants to neurodegenerative phenotypes, including amyotrophic lateral sclerosis, frontotemporal dementia, spastic ataxia, and recently, an isolated congenital myopathy, the full phenotypic and genotypic spectrum of *TUBA4A*-related disorders remains incompletely characterised.

In this multi-centre study, we identified one previously reported and 12 novel *TUBA4A* missense variants in 31 individuals from 19 unrelated families. Remarkably, individuals in 17 families presented with a myopathy without any CNS involvement or history of such disease. In the remaining two families, we observed probands with cerebellar ataxia and epilepsy accompanying proximal and axial muscle weakness along with protein aggregation. The coexistence of neuromuscular and neurodegenerative features with protein aggregation defines a multisystem proteinopathy. These two families thus establish the first association between *TUBA4A* and multisystem proteinopathy.

Our cohort exhibited diverse genotypes and inheritance patterns: four families demonstrated autosomal dominant transmission through heterozygous variants in *TUBA4A*, three probands had recessive inheritance due to homozygous variants, while the respective heterozygous carriers were asymptomatic; five probands carried *de novo* variants, and nine probands with heterozygous variants were classified as sporadic cases. Clinical phenotypes ranged from mild to severe myopathy, predominantly affecting the axial and paraspinal muscles. We observed a range of disease onset, from congenital to late adulthood. Creatine kinase levels were variable, ranging from normal to highly elevated. Cardiac function remained preserved across the cohort.

Muscle biopsies showed heterogenous myopathic changes, including myofibre size variation, nemaline bodies, core-like regions, and internal nuclei. Immunohistochemical analysis revealed protein accumulations positive for TDP-43 (n=2), p62 (n=5), and TUBA4A (n=6). Complementary *in silico* and *in vitro* investigations suggested that the identified *TUBA4A* variants cause significant protein abnormalities and may differentially impact microtubule dynamics.

Correlation analyses integrating clinical severity, variant location, and mechanistic readouts further demonstrated that domain specificity within *TUBA4A* influences both the pattern of muscle involvement and the extent of microtubule disruption.

Our findings establish myo-tubulinopathies as distinct clinical entities, encompassing both primary myopathies and multisystem proteinopathies with muscle involvement. This study broadens the phenotypic and genotypic spectrum of *TUBA4A*-related disorders beyond autosomal dominant or *de novo* mechanisms and neurodegenerative presentations. These results underscore the importance of considering *TUBA4A* variants in the differential diagnosis of axial myopathies and multisystem proteinopathies, regardless of central nervous system (CNS) involvement.

## Introduction

Classically, tubulinopathies were defined as microtubule disorders resulting from pathogenic variants in the genes encoding different tubulin isotypes.^1^ The term ‘tubulinopathy’ was first used to describe diseases associated with brain malformations arising from defects in cortical development and faulty neuronal migration, which are at the end of the phenotypic spectrum.^1^ However, ‘tubulinopathies’ are now an umbrella term for a wide spectrum of ciliary, zygotic arrest and early developmental failure, neurological and neurodegenerative phenotypes caused by pathogenic defects in microtubule units and associated proteins.^2–5^

Microtubules, a fundamental cytoskeletal component in all forms of life, exhibit dynamic instability—a continuous cycle of growth and shrinkage that is essential for various cellular functions, including maintaining cell shape, enabling intracellular transport, and participating in cell division.^6–8^ Microtubules are composed of different tubulin subunits encoded by several genes, resulting in various tubulin isotypes. The most common of these, α- and β-tubulin, form a noncovalent heterodimer, which serves as the building blocks of microtubules.^9,10^ In humans, nine genes encode different α-tubulin isotypes, each of which can potentially pair with any of the ten β-tubulin isotypes, contributing to microtubule diversity and functional specialisation.

Amongst the nine α-tubulin isotypes, *TUBA4A* encodes tubulin alpha 4a. Smith and colleagues modelled the *TUBA4A* variants first identified in patients with familial amyotrophic lateral sclerosis (ALS) and demonstrated that *TUBA4A* mutants have an impaired ability to form α- and β-tubulin dimers, which disrupts microtubule dynamics and stability in cell models through a dominant-negative mechanism.^11^ Later, pathogenic variants in *TUBA4A* were reported in patients with frontotemporal dementia (FTD),^12,13^ Parkinson’s disease,^14^ hereditary spastic ataxia,^15,16^ and zygotic arrest.^5,17^ Recently, a study identified a *de novo* variant in *TUBA4A* associated with an isolated congenital myopathy phenotype in two unrelated probands.^18^

The pathogenic variants observed so far in *TUBA4A* are not restricted to any domain; how these variants result in a specific phenotype remains unknown. Impaired dimerisation and microtubule instability are shared functional defects of most mutant tubulin proteins, including *TUBA4A* mutants.^11,19–22^ However, these gain-of-function pathomechanisms, primarily due to missense variants, can lead to vastly different phenotypes.^11,16^ Additionally, heterozygous protein truncating variants in *TUBA4A*, which escape nonsense-mediated decay and result in shorter proteins lacking parts of the C-terminal of TUBA4A, have also been associated with ALS and FTD phenotypes.^11,23^

In our study, we present a gamut of *TUBA4A*-related primary myopathies and multisystem proteinopathies, providing detailed clinical and functional insights. We identified 13 variants in 31 affected individuals from 19 unrelated families. We observed autosomal dominant, including *de novo*, and recessive inheritance. Disease onset ranged from congenital to late adulthood, predominantly affecting axial and proximal muscles. Notably, unlike other tubulinopathies, 17 out of 19 families neither exhibited any central nervous system impairment nor had a history of such disease. In the remaining two probands, multisystem involvement of myopathy, cerebellar ataxia and epilepsy were seen. Through *in silico*, *in vitro*, histopathological and immunohistochemical analyses, we characterise the phenotypic and molecular spectrum of *TUBA4A*-related myopathies and multisystem proteinopathies — henceforth referred to as ‘myo-tubulinopathies’.

## Materials and methods

### Patients and clinical examinations

All probands and affected members underwent clinical neuromuscular and neurological examinations. The inclusion criteria for this cohort were, a presumptive diagnosis of myopathy, absence of pathogenic variants in known neuromuscular disease-causing genes and the presence of a rare coding variant in *TUBA4A*, with a minor allele frequency (MAF) < 1% in gnomAD (v2.1.1/v3.1.2), identified through diagnostic or research sequencing. Only individuals with exome or genome sequencing data were included, ensuring that variants in *TUBA4A* were analysed. We collected blood and/or saliva samples for DNA extraction from affected and unaffected members from 19 families. Muscle imaging was obtained as part of the routine diagnostic work-up and was available for inclusion in this study for some patients, with consent. Electrophysiological examination results, including nerve conduction studies and needle electromyography (EMG), as well as serum creatine kinase (CK) measurements, were obtained for most patients. Aggregated clinical data was available for 21 patients. The studies were conducted per the Declaration of Helsinki, and ethical approval was obtained through institutional review boards (Supplementary Data 1).

### Muscle biopsy and immunohistochemical studies

Muscle biopsies were obtained from a subset of affected patients (*n*=13). Routine muscle histopathological studies were performed, including hematoxylin and eosin (H&E) staining, modified Gomori trichrome staining, and NADH tetrazolium reductase (NADH-TR) staining.^24^ DAB immunostaining was performed on muscle biopsy specimens from six patients and one control individual using mouse monoclonal anti-myotilin (clone RSO34, 1:20, LEICA Biosystems Newcastle Ltd, UK) and mouse monoclonal anti-desmin (clone D33, 1:70, Richard-Allan Scientific, USA), with mouse ExtrAvidin Peroxidase Staining Kit (EXTRA2, Merck KGaA, Darmstadt, Germany). Microscopic images were obtained using a NIKON ECLIPSE Ci microscope equipped with an OLYMPUS ColorView II camera.

Additional staining for aggregate pathology was performed on patient samples (Patients 5, 8, 9, 11, 12, and 24, *n = 6*). TUBA4A immunostaining was performed using a rabbit monoclonal anti-TUBA4A antibody (clone EPR13477(B), 1:100, Abcam, UK) after heat-mediated antigen retrieval with citrate buffer (pH 6.0) for 8 min at 91°C. SQSTM1 (p62) immunostaining used mouse monoclonal anti-SQSTM1 antibody (clone D-3, 1:200, Santa Cruz Biotechnology, Inc, USA). TARDBP (TDP-43) staining was performed using a rabbit polyclonal anti-TDP-43 antibody (1:1,000, Proteintech, Japan) on two biopsies (Pat.12 and Pat.24) after heat-mediated antigen retrieval with TE buffer (pH 9.0) for 8 minutes at 95°C.

### Electron microscopy and quantification analysis

Ultrathin resin sections (70–80 nm) were prepared for electron microscopy from patients 8, 9, 25 and 26 and two healthy control individuals and examined using a FEI Tecnai Spirit Transmission Electron Microscope operating at 120 kV. Electron micrographs were obtained using the Gatan Digital Micrograph (Gatan, Inc, USA).

For microtubule quantification, ultrathin resin sections from Pat.8 and Pat.9 were used. Individual microtubules were manually traced in longitudinal and cross-sectional profiles, and diameter measurements were extracted from calibrated EM images. Group-level comparisons between patients and controls were performed using standard statistical tests, with details in Supplementary Data 1.

### DNA sequencing and analysis

Targeted gene panels, short-read exome sequencing, and genome sequencing were performed in probands and available relatives, with variant confirmation and segregation assessed by Sanger sequencing. Single-nucleotide variant and indel analyses were conducted using *seqr*, RD-Connect GPAP, and in-house pipelines with standard population frequency filter: MAF < 1% in gnomAD (v2.1.1/v3.1.2/v4.1.0).

### *TUBA4A* variant analysis and interpretation

All variants were annotated to the *TUBA4A* MANE Select transcript (NM_006000.3/ENST00000248437.9) and reassessed against population frequencies in gnomAD v4.1.0. Two high-frequency, likely benign variants were included as population controls. Variant interpretation followed ACMG/AMP guidelines and was refined using VarSome, with details provided in Supplementary Data 2.

### Genotype-phenotype correlation analysis

Clinical severity, including distal and proximal limb weakness, presence or absence of axial weakness, facial weakness, respiratory involvement and ptosis were correlated with location of *TUBA4A* variants. Details are mentioned in Supplementary Data 1.

### *In-silico* modelling of *TUBA4A* variants

Computational analyses were performed to assess evolutionary conservation, predict the impact of missense substitutions on protein stability, and evaluate aggregation propensity. Free-energy changes (ΔΔG) were estimated using FoldX, and aggregation tendencies were assessed using Aggrescan4D, using AlphaFold-derived TUBA4A structures as input. Structural visualisation and *in silico* mutagenesis were performed in PyMol, with reference to available tubulin–kinesin complex structures. Detailed workflows, parameter settings, and statistical approaches are provided in Supplementary Data 1.

### *In vitro* over-expression of *TUBA4A* variants in COS1 cells

Wild-type and mutant TUBA4A constructs were generated by site-directed mutagenesis and transiently expressed in COS1 cells to assess subcellular localisation and microtubule morphology. Immunofluorescence staining and quantitative assessment of microtubule integrity were performed 48 hours post-transfection. Full experimental procedures, including cloning strategy, cell culture conditions, antibodies, imaging settings, and statistical methods, are detailed in Supplementary Data 1.

### Correlation between genotype, *in silico* and *in vitro* observations

To examine relationships between variant position, predicted molecular effects, and cellular phenotypes, we performed correlation analyses integrating FoldX stability estimates, Aggrescan4D-derived aggregation scores, microtubule morphology readouts, and domain location. Because variables were a mixture of ordinal and non-normally distributed measures, associations were evaluated using rank-based methods, with analyses involving morphology restricted to variants tested experimentally. Detailed scoring approaches, variable definitions, statistical procedures, and partial correlation models are provided in Supplementary Data 1.

## Results

### Clinical features in myo-tubulinopathies

**Figure 1:**
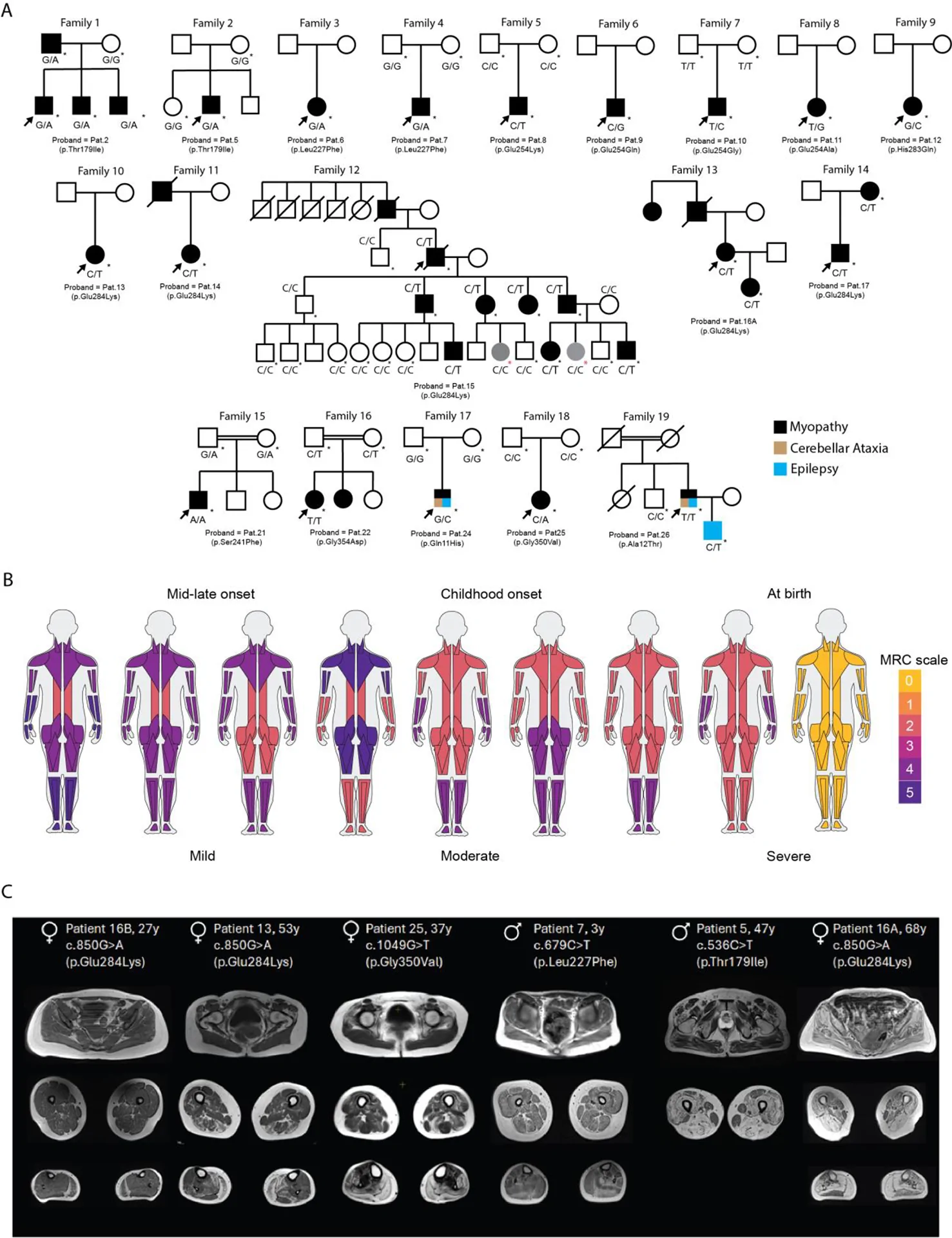
*TUBA4A*-related myopathy presents with variable patterns of muscle weakness, most consistently affecting axial muscles. **A**) Pedigrees of all 19 families included in this study. Probands are marked with a black arrow and individuals from who DNA sample was available and genotypes are marked with ‘*’. Individual families are annotated with their proband IDs and corresponding TUBA4A substitutions. Genotypes shown beneath individuals denote the *TUBA4A* variant (gDNA). In family 12, two younger individuals are coloured in grey and marked with red *. These individuals are being followed up for their reported affections status (Supplementary Data 1). In families 17 and 19, affection status is indicated in black (myopathy), brown (cerebellar ataxia), and blue (epilepsy) representing the multisystem involvement. **B**) Spectrum of muscle weakness observed in patients with variants in *TUBA4A*. MuscleViz (https://muscleviz.github.io) was used to visualise the weakness using the MRC scale for muscle strength. Muscle weakness patterns and age of onset observed in our myo-tubulinopathy cohort are shown by the body diagrams. **C**) Axial T1-weighted MR images of muscles of the gluteal region, thighs, and calves from six patients with *TUBA4A* variants, arranged from left to right in order of increasing disease severity. Each panel includes the patient’s sex, age at imaging, and specific *TUBA4A* variant.

A summary of clinical findings from 21 affected individuals is provided in Table 1. Detailed clinical descriptions of families and probands included in this study are provided in the Supplementary Data 1. Pedigrees of the families included in this study are shown in Fig. 1A.

In this cohort, the mean age of onset was 15.1 years (range: 0–60 years). Four patients had onset of neuromuscular symptoms at birth. Two patients died during the first year of life (Pat.9 and Pat.10). Within the rest of the cohort, 38% (*n*=7/18) were non-ambulant.

Most patients exhibited mild to moderate proximal and distal muscle weakness with axial involvement. Proximal muscles were generally more affected, though distal weakness, particularly in the lower limbs, was common among non-ambulant individuals. Over half of the cohort retained near-normal distal strength, whereas a smaller subset showed marked distal weakness associated with severe functional limitation. (Table 1).

A representation of muscle strength and weakness in our cohort, based on the MRC scale, is shown in Fig. 1B. Axial muscle weakness was a consistent feature in our cohort (18/19). Respiratory muscle weakness was observed in most cases (79%). Nearly half of the patients also showed facial muscle involvement, and a smaller proportion (25%) presented with ptosis. Notably, ophthalmoplegia was identified in two individuals, and ophthalmoparesis in one, an uncommon finding in myopathies. An asymmetrical muscle weakness pattern was observed in 9% of patients. Two patients (Pat.10 and Pat.23) showed severe dysphagia, while one patient (Pat.6) presented with episodic dysphagia (Supplementary Data 1). Serum CK data was available for 21 individuals in the cohort, and three of these patients had normal CK. The mean level was 1,469 IU/l (range: 143–2,794 IU/l). For patients where muscle MRIs were available (Fig. 1C), fatty degeneration of gluteus minimus and erector spinae and often preserved gluteus maximus muscles were observed.

In 17 out of 19 families, at the last assessment, no clinical signs or history of a neurodegenerative disease including hereditary spastic ataxia, ALS, dementia or Alzheimer disease were observed or stated. However, in two sporadic patients (Pat.24 and Pat.26) from different families, we observed multisystem involvement presenting as cerebellar ataxia and/or epileptic seizures along with a myofibrillar myopathy with prominent axial muscle weakness.

### Muscle pathology and protein accumulation pattern in myo-tubulinopathies

**Figure 2:**
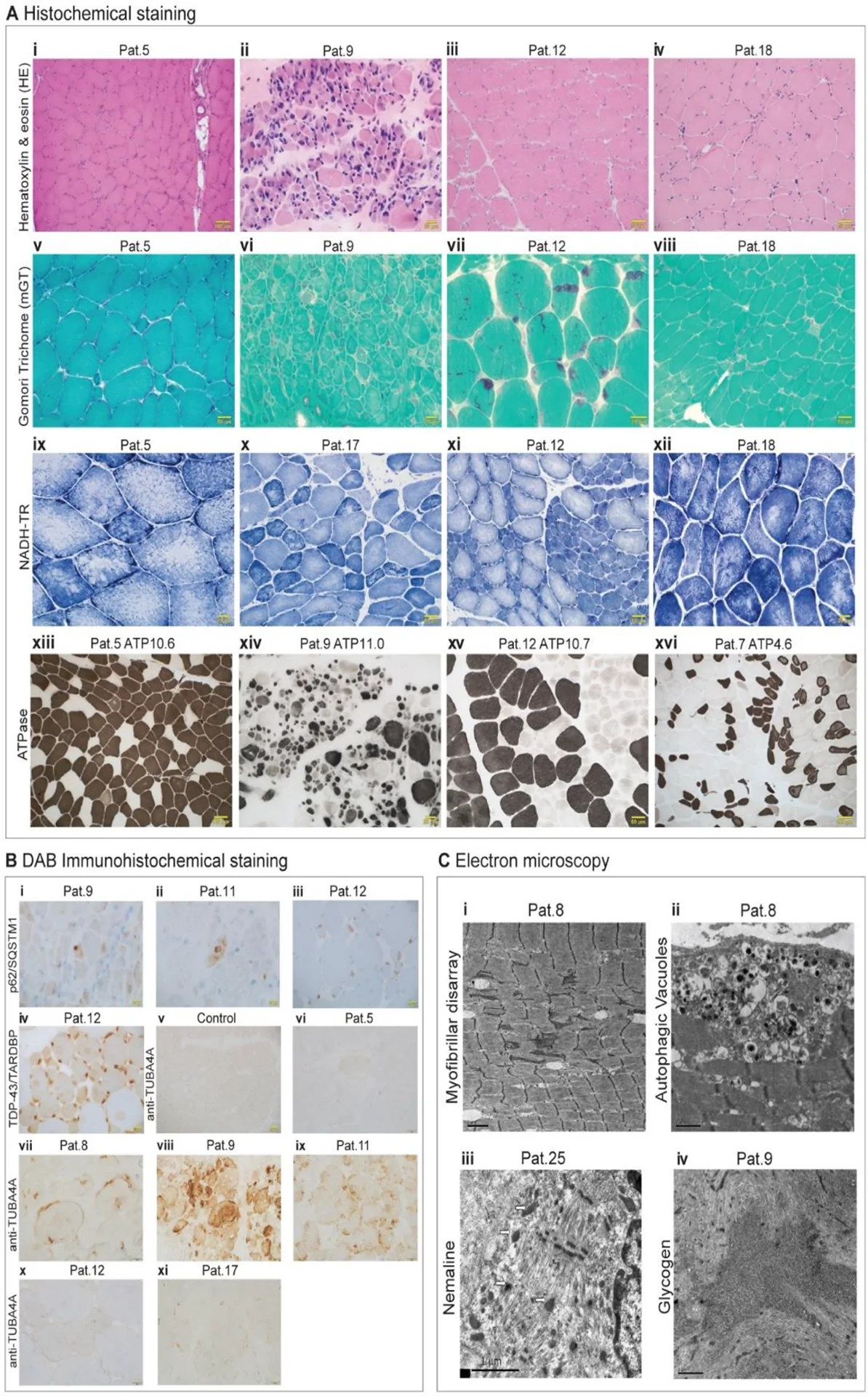
*TUBA4A*-related myopathy shows a spectrum of histological, immunohistochemical and ultrastructural findings. **A) (i-xvi)** Histochemical staining of patient muscle biopsies: **i-iv)** Hematoxylin & eosin (H&E) stain showing myofibre size variation, small angular myofibres, internal nucleation and endomysial fibrosis. **v-viii)** Modified Gomori trichome (mGT) staining showing presence of Nemaline bodies, spheroid or cytoplasmic bodies and occasional rimmed vacuoles. **ix-xii)** NADH-TR staining showing myofibre type and marked atrophy of type 1 myofibres along with minicores, large cores and moth-eaten myofibres. xiii-xvi) ATPase staining showing variability in myofibre size and atrophy, type 1 myofibre predominance and immature myofibres. **B) (i-xviii)** DAB Immunohistochemical staining of patient and control muscle biopsies: **i-iii)** Increased immunoreactivity for p62/SQSTM1. **iv)** TDP-43/TARDBP staining showed abnormal cytoplasmic accumulation. **v-xi)** Staining for anti-TUBA4A also showed variable immunoreactivity and distribution of TUBA4A across different patient muscle biopsies compared with control muscle sample at 40X magnification. **C**) **(i-vi)** Electron microscopy of patient muscles: **i-ii)** Ultrastructural analysis of Pat.8 muscle biopsy showing myofibrillar disarray and autophagic vacuoles. **iii-iv)** Pat.25 showed presence of nemaline bodies while glycogen accumulation is seen in Pat.9.

Histopathological findings, where detailed data was available (*n*=13), are summarised separately in Table 2, all muscle biopsies showed marked variation in myofibre size. Otherwise, there were no unifying histopathological hallmarks across the cohort. Eleven biopsies exhibited internally located nuclei, five showed predominance of type I myofibres and nine revealed endomysial fibrosis (Fig. 2A). Additionally, autophagic vacuoles and myofibrillar disorganisation were observed in muscle biopsies from four patients (Table 2).

To further investigate accompanying proteinopathy features, immunohistochemical analysis was performed in a subset of patients. All examined cases (*n*=5) demonstrated increased immunoreactivity for p62/SQSTM1 staining (Fig. 2B-i–iii, Table 2, Supplementary Data 1), indicating enhanced accumulation of this protein in myofibres. Phosphorylated TDP-43 accumulations were pronounced in two biopsies that were assessed (Fig. 2B-iv, Table 2, Supplementary Data 1). Similarly, anti-TUBA4A staining revealed variable levels of TUBA4A-positive accumulations across the six patient biopsies compared with control muscle, which may reflect variant-dependent differences in TUBA4A expression or accumulation (Fig. 2B-v–xi).

Additionally, a subset of patients also exhibited peculiar ultrastructural features. Nemaline bodies were identified in three patients (Pat.12, Pat.25 and Pat.26, Fig. 2A-vii, Fig. 2C-iii). Other rare features included spheroid bodies, multiminicores, moth-eaten myofibres, abnormal glycogen accumulation, and small angular myofibres (Fig. 2A, 2C).

### Identification of variants in *TUBA4A*

**Figure 3:**
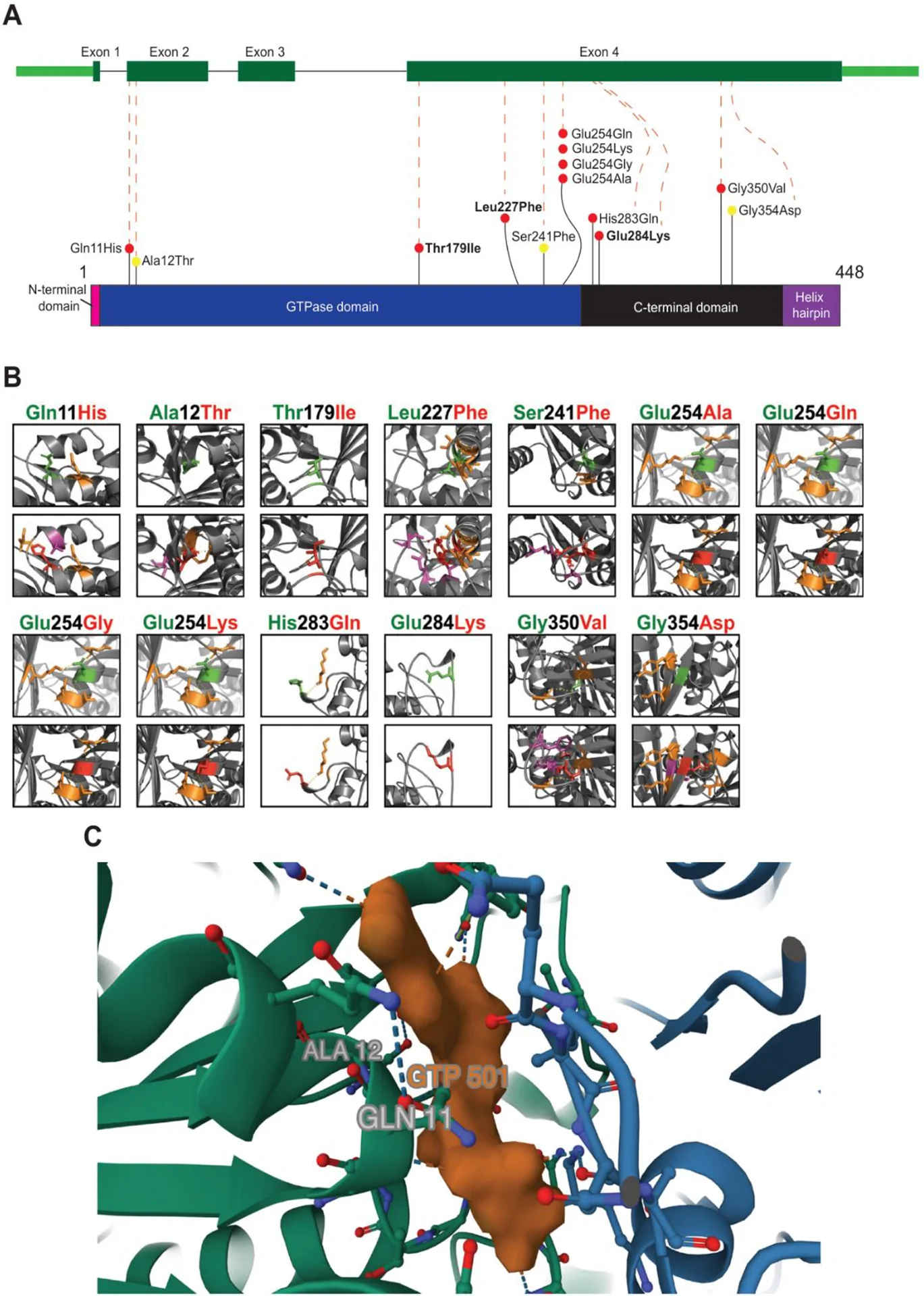
Structural mapping and three-dimensional modelling of myopathy-associated *TUBA4A* variants. **A)** Gene model of *TUBA4A* and 2D model of TUBA4A protein, showing the position of different myopathy variants (indicated as ‘coloured lollipops’) observed in our cohort. p.(Thr179Ile), p.(Leu227Phe) and p.(Glu284Lys) are in bold to indicate being observed in multiple families. p.(Ala12Thr), p.(Ser241Phe) and p.(Gly354Asp) are indicated with a ‘yellow lollipop’ to indicate homozygosity. Domain information for TUBA4A protein is obtained from Interpro-CATH gene3D. Exon 1 codes for the initiation site – methionine which forms the N-terminal domain. Thereafter, 2–268 amino acids form the GTPase domain, 269–383 form the C-terminal domain and 384–448 form the Helix hairpin bin. All our observed variants are in the GTPase domain and the C-terminal domain. **B)** The effect of identified missense variants on three-dimensional TUBA4A structure. Wild-type residues are coloured green and mutant residues are coloured red. Residues with which the mutant variant shows steric hindrance are purple. Residues which have polar contacts with the wild-type or variant residues are orange. **C)** A snapshot of mouse TUBA4A-TUBB2A with kinesin (PDB: 8IXF) using Mol*. The GTP molecule (orange) is shown along with TUBA4A (green) and TUBB2A (blue). Gln11 and Ala12 residues are labelled close to the tubulin catalytic GTP site. Gln11 is shown interacting with the GTP molecule.

Within the cohort we identified 13 different missense *TUBA4A* variants in 19 families. A representation of the observed variants in *TUBA4A* is depicted in Fig. 3A. Autosomal dominant (*n*=4 families), *de novo* (*n*=5 probands) and autosomal recessive (*n*=3 probands) variants were observed. In nine sporadic cases with heterozygous variants the mode of inheritance could not be clarified due to lack of additional familial samples. At least in one family, the deceased father of the proband (Pat.14) was indicated as affected. Aggregated genetic findings are summarised in Table 3. Interestingly, in four unrelated cases, four different heterozygous variants were identified which impacted the same amino-acid residue, Glu254, resulting in substitutions p.(Glu254Gly) and p.(Glu25Lys), confirmed to have arisen *de novo* in the probands, and p.(Glu254Ala) and p.(Glu254Gln) in two sporadic cases. Two of these variants, p.(Glu254Gln) and p.(Glu254Gly) were identified in the patients who died within the first year of life.

One heterozygous variant, p.(Glu284Lys), was identified in three unrelated families and two sporadic cases, with a total of 13 affected individuals harbouring this variant. In two probands, the same substitution, p.(Leu227Phe), was observed, one confirmed to be *de novo* and the other in a sporadic case. The p.(Leu227Phe) substitution was recently identified to have arisen *de novo* in two myopathy probands in China.^18^

Three *TUBA4A* variants were observed in homozygosity in our cohort. Firstly, the two families, Pat.21 and the siblings Pat.22 and Pat.23 with the homozygous variants, p.(Ser241Phe) and p.(Gly354Asp), respectively, had disease onset in the first year of life but this was not fatal in the first year. The parents of these three patients were heterozygous carriers and did not manifest any symptoms of neuromuscular disease. However, no additional investigations (e.g., muscle MRI, EMG, or biopsy) were performed in these unaffected carriers due to geographic and logistical constraints. We therefore cannot exclude the possibility of subclinical manifestations or incomplete penetrance, and this remains a limitation of the study.

In the family of Pat.26, the proband, with an age of onset at 60 years, was observed to be homozygous for variant p.(Ala12Thr). His son was a heterozygous carrier of the variant and at last clinical assessment had idiopathic epilepsy without any neuromuscular issues. A healthy brother of the proband had a normal genotype (Supplementary Data 1).

Nine variants were in the guanosine triphosphate (GTP)-ase domain, and the remaining four variants were in the C-terminal domain (Fig. 3A). All identified variants were in exon 4, except for p.(Gln11His) in Pat.24 and p.(Ala12Thr) in Pat.26 which were within exon 2 near the GTP-binding site (Fig. 3C). All 13 variants were absent in population databases including gnomADv4.1.0 (Table 3, Supplementary Data 2).

### Genotype–phenotype correlations reveal domain-specific severity patterns in myo-tubulinopathies

Correlation analyses showed that variants within the GTPase domain showed stronger negative correlations with distal upper (*r* = −0.56) and distal lower limb weakness (*r* = −0.40), consistent with greater clinical severity compared to variants in the C-terminal (*r* = +0.37, +0.27) or GTP-binding site (*r* = +0.31, +0.21) (Supplementary Data 1). Domain-wise correlations showed that GTPase-domain variants more frequently co-occurred with facial weakness (*r* = +0.70) and ptosis (*r* = +0.41) compared with other domains. CK levels showed minimal domain-related association (*r* = −0.21 to +0.36), and overall severity correlated negatively only with GTPase-domain variants (*r* = −0.40). Axial weakness was present in almost all affected individuals, with only one case lacking axial involvement and one lacking sufficient clinical information; this limited variability prevented a meaningful correlation analysis.

Importantly, both individuals with multisystem proteinopathy (Pat.24 and Pat.26) carried variants in or near the GTP-binding site, whereas all other domains were associated with isolated myopathy, suggesting that perturbation of GTP-binding dynamics may contribute towards multisystem involvement.

### Structural modelling and aggregation analyses reveal variant-specific molecular impacts on TUBA4A

Multiple sequence alignments were performed for TUBA4A orthologs across 10 non-human species (Supplementary Data 1), and all human alpha tubulins (Supplementary Data 1), anchored to the human TUBA4A protein sequence. All the identified myo-tubulinopathy variants affected highly conserved amino acid residues.

The wild-type TUBA4A protein was predicted by AlphaFold2. The ‘mutagenesis’ function on PyMol was used to predict changes in protein structure and/or folding upon incorporating substituted amino acid residues into the sequence (Fig. 3B). Most substitutions showed a change in steric hindrance with nearby or distant amino acid residues. Of note, the two variants observed as *de novo*, p.(Leu227Phe), p.(Gly350Val) and the homozygous variant p.(Gly354Asp) showed the most substantial changes to polar contacts and steric hindrance upon substitution. Thus, we predicted that these substitutions could affect protein stability.

**Figure 4:**
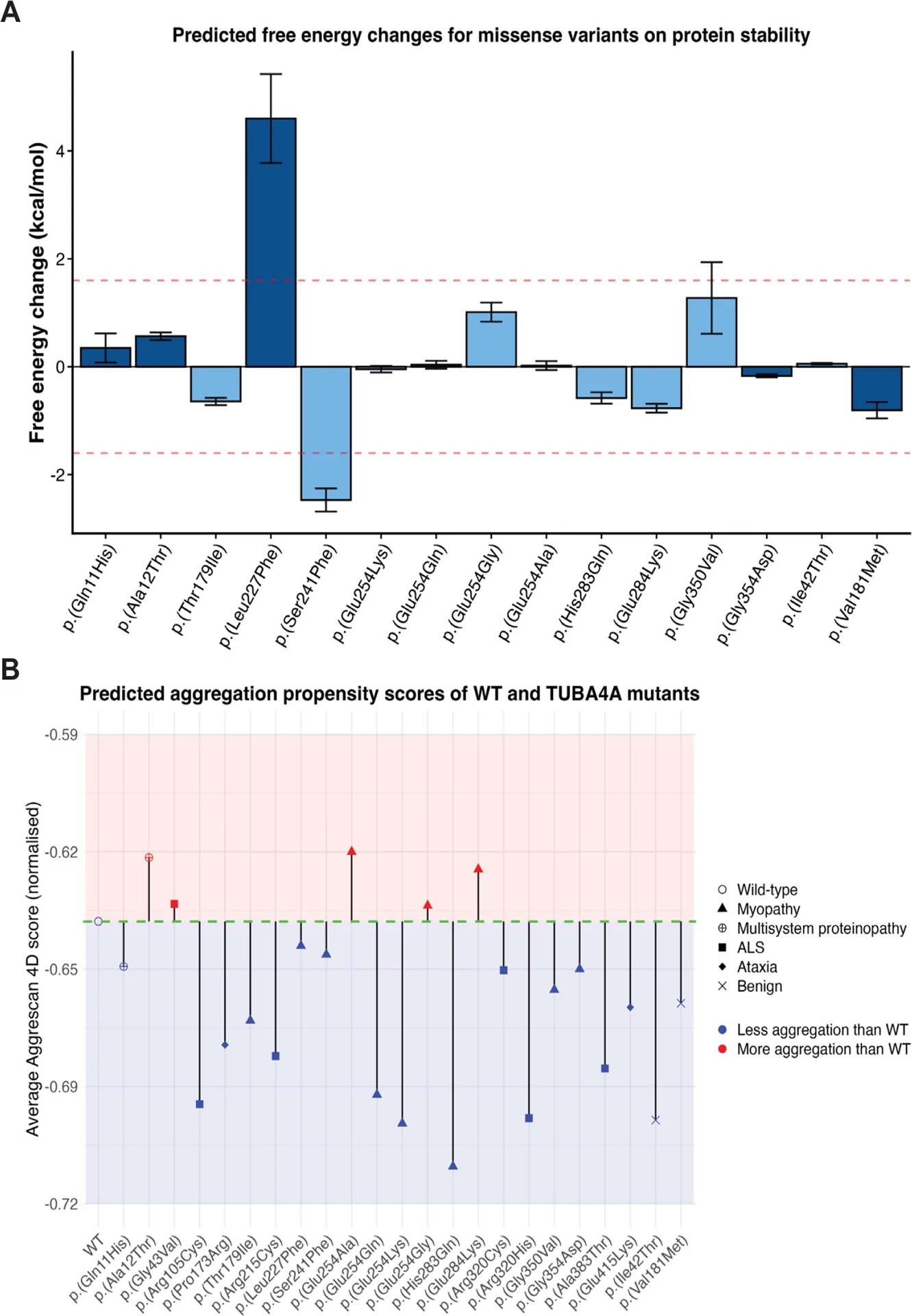
*In silico* assessment of protein stability and aggregation propensity suggests distinct biophysical consequences associated with different *TUBA4A* variants. **A)** Predicted free energy changes (ΔG_MUT_ – ΔG_WT_) for missense variants on protein stability. Calculations were performed for variants within very high confidence regions (pLDDT > 90, dark blue bars) or confident regions (70 < pLDDT < 90, light blue bars) of the AlphaFold2-generated TUBA4A protein. Data are given as mean ± s.d. The red dashed lines indicate conservative thresholds (±1.6 kcal/mol). **B)** Aggrescan 4D prediction plot recreated with Aggrescan 4D scores showing aggregation propensity of wild type TUBA4A and TUBA4A variants including 13 variants identified in the present study for myopathy and multisystem proteinopathy, six variants associated with ALS, two variants previously published in association with ataxia and two benign variants. The x-axis represents the residue position, and the y-axis represents the normalised average aggregation propensity scores calculated by Aggrescan 4D.

Free energy change analyses showed that, of the identified substitutions, only the p.(Gln11His), p.(Ala12Thr), p.(Leu227Phe), and p.(Gly354Asp) were in the very high confidence regions (pLDDT > 90) of the AlphaFold2-predicted protein structure. FoldX-calculated free energy changes for these substitutions indicated that only the p.(Leu227Phe) substitution has a significant decrease in free energy > 1.6 kcal/mol, suggesting a protein destabilising effect with high-confidence (Fig. 4A). This aligns with the previously observed steric hindrance caused by p.(Leu227Phe) (Fig. 3B).

In contrast, the p.(Ser241Phe) substitution showed a significant increase in free energy. This may predict a stability increase, but it cannot be excluded that the variants could cause a deleterious effect to protein folding or stability by another unknown mechanism.

Additionally, we used the experimentally validated model of mouse TUBA4A-TUBB2A dimer with kinesin (PDB: 8IXF) to visualise variant impact on the GTP binding site. In the α/β-tubulin heterodimer, both subunits can bind GTP; however, only the β-tubulin subunit can hydrolyse GTP to GDP, while the GTP bound to α-tubulin (TUBA4A) remains non-exchangeable and structurally stabilising. The Gln11 and Ala12 residues in TUBA4A are in proximity to the non-exchangeable GTP-binding site, with Gln11 directly interacting with the nucleotide (Fig. 3C). We predict that substitutions at these positions, which alter the residue charge or polarity, may impair GTP binding and thereby destabilise the TUBA4A structure or its interaction with the β-tubulin, thus affecting the microtubule dynamics, an underlying pathomechanism of different tubulinopathies.

Given that structural destabilisation alone may not fully capture the pathogenic potential of the identified variants, we subsequently investigated the aggregation propensity of TUBA4A variants, as stability and aggregation are distinct yet interconnected properties. While FoldX evaluates thermodynamic stability of the native protein fold, aggregation analysis specifically examines the potential for proteins to form multi-protein assemblies with potentially non-native conformations.

A variant may significantly destabilise the structure (high ΔΔG in FoldX) without promoting aggregation if it lacks aggregation-prone motifs or is managed by chaperones.^25,26^ Conversely, even stable variants can expose or create aggregation-prone regions, altering cellular behaviour.^27^ By evaluating both factors, we aimed to clarify how TUBA4A substitutions might contribute to the pathobiology through distinct molecular mechanisms.

Aggrescan 4D predicts whether individual residues are aggregation prone (positive score, >0), have increased solubility (negative score, <0) or no change in solubility (score=0) (Supplementary Data 2). The dynamic mode also predicts the change in interacting residues and the aggregation propensity of the whole protein. Thus, the average aggregation score represents the average of all 448 residues of TUBA4A in both wild-type and each variant protein. All tested variants, including the 13 identified variant residues in our study and the eight previously observed ALS/ataxia-causing variants alter the aggregation propensity of the protein in a variable manner (Fig. 4B, Supplementary Data 2). As expected, the two likely benign variants included in this study did not exhibit protein-destabilising effects or reduced solubility compared to wild-type TUBA4A (Fig. 4B, Supplementary Data 1).

These findings suggest that distinct pathogenic variants, differing in predicted stability and aggregation propensity, may modulate TUBA4A behaviour through diverse mechanisms.

### *In-vitro* studies show variant-specific disruption of TUBA4A localisation and microtubule morphology

**Figure 5:**
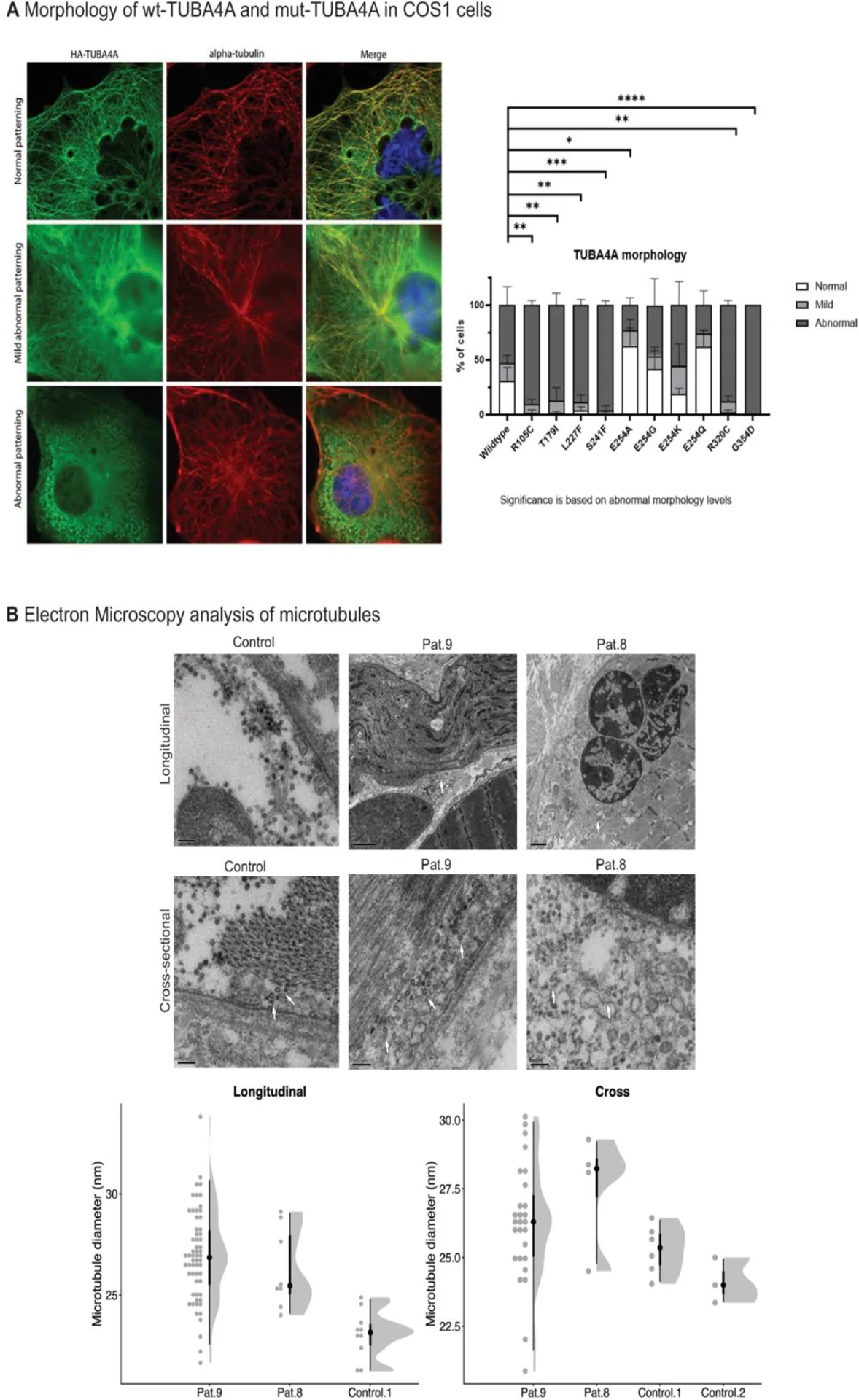
*TUBA4A* variants disrupt microtubule morphology in cellular models and patient muscles. **A)** Molecular physiology of cytoskeleton in COS1 cells overexpressed with TUBA4A-wildtype (wt) and TUBA4A variants. Morphology ranged from normal, mild to abnormal cytoskeleton. As compared to TUBA4A-wt, previously reported disease-causing variants Arg105Cys (ALS) and Arg320Cys (ALS) showed abnormal cytoskeleton structure. Additionally, the myopathic variants identified in our study, Thr179Ile, Leu227Phe, Ser241Phe and Gly354Asp showed abnormal cytoskeletal patterning. Conversely, Glu254Ala, Glu254Gly, Glu254Lys and Gln254Gln showed normal to mildly abnormal cytoskeletal patterning. **B)** Electron microscopy analysis of microtubules in control muscles and patient muscles showing statistically significant increase in microtubule diameter in patient muscles as compared to controls. Each point represents an individual microtubule measurement from patient (Pat.8, Pat.9) or control (Control1, Control2) muscle. Half-violin plots show the distribution with median and 50/95% intervals. Longitudinal measurements were significantly increased in patients vs controls (26.8 vs 23.1 nm; Δ = 3.79 nm, 95% CI 2.82–4.75; Welch’s t-test p = 8.8 × 10^−8^). Cross-sectional diameters showed a smaller but significant increase (26.4 vs 24.9 nm; Δ = 1.53 nm, 95% CI 0.49–2.58; p = 0.0055).

To experimentally evaluate the effect of the missense substitutions on intracellular localisation of TUBA4A, COS1 cells were transfected with either WT or TUBA4A variants: p.(Arg105Cys), p.(Thr179Ile), p.(Leu227Phe), p.(Ser241Phe), p.(Glu254Ala), p.(Glu254Gln), p.(Glu254Lys), p.(Glu254Gly), p.(Arg320Cys) and p.(Gly354Asp). Overexpression of wild-type TUBA4A led to an abnormal TUBA4A localisation pattern in ~53% of the cells. A similar result was seen for p.(Glu254Gly) (~47%) and p.(Glu254Lys) (~56%), while p.(Glu254Ala) and p.(Glu254Gln) showed less abnormal cells, ~23% and ~26% respectively. A near complete abnormal localisation pattern was observed in ~87–100% of the cells (Fig. 5A) over-expressing p.(Thr179Ile), p.(Leu227Phe), p.(Ser241Phe), p.(Arg320Cys) and p.(Gly354Asp) localisation. A limitation of this approach was that microtubule morphology was scored manually based on a set of images, which may introduce observer bias.

### Genotype–mechanistic correlations highlight domain-specific effects on microtubule organisation

Mechanistic correlation analyses revealed that microtubule morphology was most strongly influenced by the affected TUBA4A domain. Among variants for which morphology data were available (*n*=10), C-terminal substitutions showed markedly higher morphology severity scores than GTPase variants, yielding a strong positive correlation between domain and microtubule disruption (*ρ* = 0.52). Predicted effects on protein stability (FoldX ΔΔG) showed only a weak association with morphology (*ρ* = −0.25), which remained modest after adjusting for domain (partial *ρ* = −0.25). Aggregation propensity demonstrated a modest negative correlation with morphology severity (*ρ* = −0.27), although domain again accounted for much of this effect (partial *ρ* = −0.29). Across all variants (*n*=15), domain showed minimal association with ΔΔG or aggregation propensity, indicating that these computational measures do not segregate cleanly by the location of the variant in a particular domain (Supplementary Data 1).

Taken together, these data indicate that microtubule disorganisation is driven primarily by the functional domain affected, particularly the C-terminal tail, rather than by the predicted magnitude of protein destabilisation or aggregation propensity, underscoring domain-specific mechanisms of pathogenicity in variants in *TUBA4A*-related disorders.

### Microtubule thickening in myo-tubulinopathies

To investigate potential ultrastructural differences in microtubules between patients and age matched controls, we performed additional electron microscopy investigations of patient muscle biopsies from Pat.8 (p.(Glu254Lys), 4-year-old male) and Pat.9 (p.(Glu254Gln), 4-month-old male) and compared them to control biopsies (5-year and 2-year-old males). This analysis revealed significantly thicker microtubules myofibres in patient muscle compared to age-matched healthy controls (*p* < 0.0001) (Fig. 5B, Supplementary Data 1).

### Reassessment of identified *TUBA4A* variants

Based on the genetic data, *in silico* evidence, and findings from the *in vitro* assay it was possible to reclassify most of the variants in the myo-tubulinopathy cohort using the ACMG/AMP guidelines (Table 3, Supplementary Data 2).^28^ Accordingly, classification of eight variants could be upgraded to ‘pathogenic’ using additional ACMG/AMP criteria, while there was sufficient evidence to suggest upgrading four variants to ‘likely pathogenic. One variant had insufficient evidence and remained as VUS (Table 3, Supplementary Data 2). Raw and revised classification and assertion criteria details can be seen in Supplementary Data 2.

## Discussion

In this multi-centre study, we identified 13 different missense variants in *TUBA4A* causing a myopathy, with childhood onset of disease and prominent weakness in proximal and axial muscles being the most frequent clinical presentation. Consistent with our methods of ascertainment, which were largely focused on myopathic cases from neuromuscular disease cohorts, 17/19 families did not have a recorded history of CNS disorders, thus this represents an important phenotypic expansion of *TUBA4A*-related disease to primary skeletal muscle conditions. Two probands presented with multisystem involvement: a mild myofibrillar myopathy along with cerebellar ataxia, cerebellar atrophy and epileptic seizures. These two cases thus represent the first incidence of an MSP phenotype associated with pathogenic variants of *TUBA4A*. Both MSP associated variants occurred in exon 2 of *TUBA4A*, whereas the other 11 variants associated with pure myopathic phenotypes were clustered in exon 4. Three probands harboured homozygous variants in *TUBA4A consistent with the observed recessive phenotypes*. Of these, two probands presented with severe, early-onset axial myopathy, while the third displayed a later-onset multisystem phenotype.

In general, tubulinopathies are either predominantly autosomal dominant (AD) or *de novo*/sporadic conditions and are rarely autosomal recessive.^29^ In our cohort, we observed four families with autosomal dominant inheritance. Twelve cases had sporadic occurrence of a skeletal muscle phenotype where five probands had *de novo* variants in *TUBA4A*, while in seven isolated cases, we could not clarify the mode of inheritance due to absence of additional familial DNA. Of the remaining families, three showed clear autosomal recessive inheritance with affected individuals homozygous for bi-parentally inherited variants.

Notably, multiple substitutions affecting the same amino acid residue resulted in similarly severe myopathic phenotypes in our cohort. Four substitutions were observed at Glu254 (Glu254Gly, Glu254Lys, Glu254Ala and Glu254Gln) in four different probands, each time resulting in a severe early onset phenotype, including two who died in the first year of life. Thus, we observed a range of clinical features including different patterns of inheritance, varying severity of muscle weakness from death in the first year of life (n=2), non-ambulation in childhood, to mild or moderate weakness in adulthood.

To explore how variant location relates to clinical presentation, we examined patterns of muscle involvement across TUBA4A domains. Variants in the GTPase domain showed the strongest associations with more severe muscle weakness (negative r values across distal UL and LL strength), whereas variants within the C-terminal domain aligned with milder presentations. Together, these patterns suggest that domain specificity meaningfully shapes both the severity and distribution of neuromuscular weakness, contributing to the heterogeneous clinical spectrum observed in this cohort.

In patient muscles biopsies, histopathological findings included characteristic features of a myopathy such as variation in myofibre size, myofibrillar disorganisation, type 1 myofibre predominance, internal nucleation, regenerating and degenerating myofibres, autophagic vacuoles (*n*=4, Table 2), nemaline bodies (*n*=4, Table 2, Supplementary Data 1), minicores and moth-eaten myofibres. Ultrastructural studies showed classical signs of myofibrillar disarray (*n*=4, Table 2). Muscle biopsies from the two MSP cases showed similar myopathic features. Owing to the severity of the clinical presentation in affected individuals from the two recessive families, muscle biopsies could not be performed for diagnostic evaluation. In our cohort, however, there were no clear unifying histopathological features. Previously Wan and colleagues^18^ showed that in *TUBA4A*-p.(Leu227Phe) related myopathy, both probands exhibited myofibres with focal myofibrillar disorganisation and rimmed vacuoles.

Variable accumulation of phosphorylated TDP-43, often considered as one of the markers of a proteinopathy, showed that different *TUBA4A* variants might result in distinct level of protein accumulations. Similarly, TUBA4A-positive accumulations were also increased in a subset of muscle biopsies compared to others and controls. Although none of the previous *TUBA4A*-related ataxia and ALS studies investigated specific autophagic disturbances in patient biopsies, Smith and colleagues^11^ showed accumulation of TUBA4A p.(Trp407Ter) in transfected primary motor neurons. Wan and colleagues^18^ showed ubiquitin-positive TUBA4A accumulations of the TUBA4A-p.(Leu227Phe) variant in transfected COS7 cells and suggested the involvement of autophagic disturbances. Interestingly, the same variant, p.(Leu227Phe), was observed in two cases within our cohort (*de novo* in Pat.7 and as an isolated sporadic occurrence in Pat.6).

Complementing these earlier findings,^18^ we also observed classical signs of disturbed autophagy, i.e. p62/SQSTM1-positive accumulations in myofibres of affected patients. However, our immunohistochemistry data together with previous studies in tubulin-related pathomechanisms^4,11^, indicate that protein accumulations may not be the sole contributor to the pathobiology of the disease.

We therefore used a range of *in silico* methods to predict and analyse the molecular impact of identified variants in *TUBA4A*. Given that tubulin variants can disrupt either tubulin monomer dimerisation or microtubule stability through a dominant-negative mechanism, these computational approaches may offer insights into the distinct ways each variant might contribute to disease pathology.

3D modelling studies indicated that p.(Leu227Phe) showed considerable changes to polar contacts and steric hindrance upon substitution, which could affect protein stability. Further, our FoldX calculations also showed that p.(Leu227Phe) exhibited significantly elevated free energy changes (threshold 1.6 kcal/mol), indicating substantial protein destabilisation. Our *in vitro* studies in COS1 cells showed abnormal microtubule morphology in cells transfected with the p.(Leu227Phe) variant. These observations are in alignment with Wan and colleagues^18^ who showed that the TUBA4A:p.(Leu227Phe) variant forms ubiquitin-positive TUBA4A accumulations in transfected COS7 cells. Notably, while FoldX predicted severe destabilisation, our *in-silico* aggregation propensity analysis did not suggest an increased tendency toward aggregation for this variant compared to wild-type TUBA4A.

Our integrated analysis suggests that the p.(Leu227Phe) substitution introduces a bulkier aromatic side chain in a spatially constrained region of TUBA4A, creating specific steric clashes with its neighbouring residues. These structural perturbations directly disrupt the tightly packed hydrophobic interactions that stabilise this domain, accounting for the observed significant increase in free energy. Thus, p.(Leu227Phe) contributes to pathology likely through the thermodynamic destabilisation of the protein’s native fold which may impair proper degradation of the protein resulting in improper localisation and accumulation of the mutant protein rather than through increased aggregation propensity.

The p.(Glu254Ala) and the p.(Glu254Gly) variants displayed increased aggregation propensity compared to the wild-type TUBA4A, while our FoldX calculations showed that all identified variants at this hotspot [p.(Glu254Ala), p.(Glu254Lys), p.(Glu254Gln), and p.(Glu254Gly)] have relatively minimal changes in free energy. Interestingly, *in vitro* over-expression studies revealed normal to mild morphological changes in COS1 cells expressing these four variants. This contrasts with the clinical presentation, as patients harbouring variants at this position exhibited the most severe phenotypes in our cohort. Notably, muscle biopsies of Pat.8 with p.(Glu254Lys), Pat.9 with p.(Glu254Gln) and Pat.11 with p.(Glu254Ala) showed TUBA4A positive protein accumulations (Fig. 2B:vii–ix). Pat.8 and Pat.9 also showed significantly dilated microtubules in skeletal muscle compared to the controls (Fig. 5B). Again, this divergence between computational predictions, cellular phenotypes, and clinical severity highlights the complex nature of the effects of these variants and the limitations of *in-silico* approaches and *in vitro* over-expression assays. While variants at this hotspot may promote aggregation, and possibly impact tubulin dimerisation, the exact pathomechanism underlying the noteworthy clinical impact of this mutational hotspot requires further investigation.

To integrate our molecular and cellular findings, we performed correlation analyses across ΔΔG predictions, aggregation propensity, and microtubule morphology. These analyses did not support discrete mechanistic clusters based on stability or aggregation alone. Instead, the variant position within TUBA4A emerged as the most informative predictor of cellular phenotype: C-terminal variants correlated with the greatest microtubule disruption, whereas GTPase-domain variants showed milder effects. In contrast, ΔΔG and aggregation scores demonstrated only weak or inconsistent relationships with morphology, indicating that domain-specific structural context plays a larger role than overall predicted destabilisation.

While these integrated *in silico* and *in vitro* analyses provide domain-level insight, we acknowledge that deeper mechanistic characterisation of individual variants was beyond the scope of this study and will be important for defining their precise pathogenic effects.

The p.(Glu284Lys) variant identified in five unrelated families and 13 affected individuals with similar mild myopathic presentation in our myo-tubulinopathy cohort, presents a notable case of phenotypic divergence. This variant has previously been reported in relation to human zygotic arrest and early developmental failure in heterozygosity.^5,30^ All cases in our cohort, harbouring this heterozygous variant, either showed autosomal dominant inheritance (Pat.15, Pat.16A, Pat.16B, Pat.17, and Pat.18) or had an undetermined inheritance (Pat.13 and Pat.14), and none of the affected females reported infertility or reproductive complications. Conversely, none of the individuals in the fertility studies^5,30^ were reported to have a neuromuscular disease (personal communication), suggesting potential tissue-specific effects of the variant. However, this pleiotropic effect is not unique to *TUBA4A*, as similar divergent phenotypes have been observed with other variants such as *TUBB3* p.Arg262His - implicated in cortical malformations, peripheral neuropathy and endocrine malfunction,^31^ and *LMNA* p.Arg644Cys, which has been variably associated with muscular dystrophy, cardiomyopathy, insulin resistance, lipodystrophy and peripheral neuropathy.^32^

In Pat.21 and the two affected siblings Pat.22 and Pat.23 there was autosomal recessive myo-tubulinopathy, associated with homozygous p.(Gly354Asp) and p.(Ser241Phe) pathogenic variants, respectively. Interestingly, these three patients also presented with fatigability. Repeat nerve stimulation (RNS) showed decrements after repetitive stimulation of ulnar nerve and accessory nerve (Supplementary Data 1). The clinical presentation of fatiguability and decrement on RNS prompted treatment with pyridostigmine. Both patients showed improved muscle function post-treatment. Pat.7 with the *de novo* p.(Leu227Phe) variant and Pat.25 with the *de novo* p.(Gly350Val) variant also showed similar fatigability. Pat.7 showed decrement of up to 17% in ulnar nerve and was prescribed salbutamol 0.1 – 0.3 mg/kg/day for 2–3 months but showed no improvement. For Pat.25 there was no decrement observed on RNS of ulnar and ocular muscle but from deltoideus (up to 22%). She also did not respond to the salbutamol treatment. This aligns well with our integrated findings that specific molecular consequences unique to each *TUBA4A* variant and their distinct downstream interactions may exist in different myo-tubulinopathies, ultimately determining the potential therapeutic approaches and outcomes.

In our cohort, 17/19 families did not show a history of a CNS disease, and the phenotype observed was primary skeletal muscle disease. However, in these patients, especially with earlier onset and the young ages within the cohort, involvement of CNS may occur at a later age.

Interestingly, in Pat.24 harbouring the heterozygous likely-pathogenic, p.(Gln11His), we observed a mild early-onset cerebellar atrophy along with a myofibrillar myopathy (MFM). In Pat.26 harbouring the homozygous VUS, p.(Ala12Thr), pathologically confirmed MFM and concomitant late-onset ataxia with cerebellar atrophy was observed.

The positively charged Gln11 residue interacts with the α-tubulin catalytic GTP site. A change to the negatively charged His at this position will potentially inhibit the GTP binding activity and hence could impact microtubule dynamics. In the α/β-tubulin heterodimer, α-tubulin has a non-hydrolysing and non-exchangeable “N-site” which binds to GTP, whereas β-tubulin has an exchangeable “E-site” where GTP can be hydrolysed into GDP.^33,34^ Recently, McFadden and colleagues reported the p.(Glu11Arg) variant in *TUBB4B*, in a syndromic presentation of hearing loss, hyperopia without retinal abnormalities, renal tubular Fanconi Syndrome and hypophosphatemic rickets. Functional studies showed that this *TUBB4B* p.(Glu11Arg) variant results in inhibition of GTPase activity due to the Arg impeding depolymerisation and hence results in hyperstability of the polymerised microtubule.^35^ The hydrophobic Ala12 residue in TUBA4A is close to the α-tubulin GTP binding site, and similarly a change to a polar uncharged Thr residue at this position might impact the GTP binding activity of the heterodimer. Notably, the Gln11His variant was identified as a heterozygous *de novo* change, whereas the Ala12Thr variant was homozygous. Despite these distinct zygosities, both variants are associated with similar MSP phenotypes, raising intriguing questions about the mechanistic convergence of their allelic presentations.

Pat.26 with the homozygous VUS p.(Ala12Thr) was previously published with a VUS in *FLNC* thought to be the cause of the myofibrillar myopathy in the proband.^36^ This *FLNC* variant (p.(Thr2419Met)) has since been reported as ‘likely benign’ in multiple submissions in ClinVar (VCV000712192.18). Therefore, with the current knowledge, it is unlikely to be the primary cause of disease in the proband, although a potential role in modulating the phenotype cannot be excluded.

From our results, we believe that these two substitutions, *TUBA4A* p.(Gln11His) and p.(Ala12Thr), are quite distinctive in terms of their clinical presentations involving multiple systems, possibly due to the unique biochemical changes to the GTP-binding site in TUBA4A.

In general, MSPs encompass a group of inherited disorders characterised by the degeneration of muscle, bone, and nervous tissues.^37^ A disease presentation can be classified as an MSP if a neurodegenerative and a neuromuscular phenotype with protein aggregations coexist in the same individual or co-segregate with a variant in the same family.^37,38^ The MSP spectrum includes clinical phenotypes such as rimmed vacuolar myopathy (RVM), Paget disease of bone (PDB), FTD, and ALS.^37^ The pathomechanisms involve disruptions in key protein clearance pathways, specifically the ubiquitin-proteasome system (UPS) and autophagy.^37^ This leads to accumulation of toxic protein aggregates, characterised by immunopositivity for key UPS and autophagy proteins, including RNA binding proteins, sequestosome 1/p62 (SQSTM1),^39^ and TDP-43.^38^

*TUBA4A* variants have been implicated in neurodegenerative diseases such as ALS,^11,40–42^ FTD,^12,13,43^ and ataxia.^15^ In our study, p62 positive accumulations are observed in all patients in whom p62 staining was performed (n = 5). Wan and colleagues also showed presence of p62 positive TUBA4A accumulations in COS7 cells transfected with TUBA4A p.L227F.^18^ The observation of an MSP phenotype in Pat.24 (p.(Gln11His)) and Pat.26 (p.(Ala12Thr)) in our cohort suggests that certain *TUBA4A* variants may result in a multisystem involvement. *TUBA4A* should be examined as a candidate gene in patients with molecularly undiagnosed MSP.

How diversity of neurodegenerative and neuromuscular phenotypes associated with TUBA4A—and tubulins more broadly arises is partly explained by the concept of the ‘tubulin code’. This concept proposes that the functional diversity of microtubules, across tissues, is regulated by the expression of distinct tubulin isotypes and a range of post-translational modifications (PTMs), including acetylation, polyglutamylation, and detyrosination.^44,45^ These PTMs fine-tune microtubule dynamics, stability, and interactions with microtubule-associated proteins, tailoring microtubule behaviour to specific cellular contexts.^46^ In skeletal muscle, where microtubules contribute to mechanical stability, nuclear positioning, and metabolic regulation, microtubule regulation is especially critical.^47^ Disruption of TUBA4A in this context may therefore have a more pronounced effect on microtubule function, directly impairing myofibre integrity and contractility.

Dodd and colleagues^4^ recently showed that the phenotypic diversity of *TUBB4B*–related disease reflects variant-specific effects on microtubule biology. Through structural and functional assays, they demonstrated that different variants map to distinct protein surfaces and perturb microtubule behaviour through separate dominant-negative mechanisms—either disrupting α/β-heterodimer assembly or incorporating into microtubules and altering their dynamics. These findings illustrate how variant position influences biochemical behaviour and clinical outcome, consistent with the regulatory logic of the tubulin code.

Our clinical and mechanistic correlations parallel the domain-specific patterns described by Dodd and colleagues. As in *TUBB4B*, where variant position mapped to distinct biochemical behaviours, we observed that *TUBA4A* variant location predicts both clinical severity and cellular phenotypes—from GTPase-domain substitutions associated with more pronounced muscle weakness to C-terminal variants producing the strongest microtubule defects in vitro. Although only two C-terminal variants had morphology data, the emerging structure–function trends are consistent with principles seen across tubulin isotypes. This supports the broader logic of the tubulin code, in which biochemical and spatial context, not the specific amino-acid change alone, shapes variant behaviour and phenotypic expression.

Importantly, the clinical and mechanistic correlations each revealed domain-specific patterns, but their relative severity differed across analyses. This divergence likely reflects the distinct biological levels captured: whole-muscle physiology versus acute cellular responses to mutant tubulin and highlights the context-dependent nature of tubulin dysfunction.

The publicly available GTEx^48^ and Human Protein Atlas^49^ data show that α-tubulin 4A is ubiquitously expressed, with particularly high levels in skeletal muscle. The broad range of disease onset, heterogeneous clinical and histopathological features, and multiple inheritance patterns observed in this cohort underscore the diagnostic challenge posed by myo-tubulinopathies. Notably, all pathogenic variants identified were missense changes, many of which would be classified as VUSs under current guidelines due to limited functional evidence and the lack of an established gene–disease framework for *TUBA4A*. This gap raises the risk that pathogenic variants remain unreported or misclassified, delaying diagnosis and appropriate clinical management.

In conclusion, this study establishes that missense variants in *TUBA4A* can cause either isolated myopathy or multisystem proteinopathy with variable clinical and histopathological features, including p62 accumulation, internal nucleation, autophagy dysfunction, nemaline bodies and myofibrillar disarray. These findings support a role for *TUBA4A* in maintaining skeletal muscle integrity through the regulation of microtubule function and dynamics. Our large, multi-centre cohort expands the phenotypic and inheritance spectrum of *TUBA4A*-related disease, with most patients showing no evidence of CNS involvement. Together, these results underscore the importance of including *TUBA4A* in genetic screening panels for unresolved myopathies and multisystem proteinopathies.

## Supplementary Material

Supplementary Table

Supplementary Data

Supplementary Author Info

Supplementary material is available at *Brain* online.

## Figures and Tables

**Table 1:** Summary of clinical features of affected individuals in our myo-tubulinopathy cohort with disease causing variants in *TUBA4A*

	Individuals with disease causing variants in *TUBA4A* (Number of patients, n=21, number of families, N=19)	
	Total	Percentage
**Inheritance (families)**		
AD	4/19	21%
AR	3/19	16%
*De novo*	5/19	26%
Sporadic^a^	7/19	47%
**Onset**		
At birth	4/21	19%
Childhood	11/21	52%
Adult and late adult (18–30)	2/21	10%
Mid or late onset (30–60)	4/21	19%
**Biological Sex**		
Male	11/21	52%
Female	10/21	48%
**Muscle weakness (MRC 4–5, 2–3, 0–1)**		
Distal UL		
MRC 4–5	11/20	55%
MRC 2–3	4/20	20%
MRC 0–1	5/20	25%
Distal LL		
MRC 4–5	11/20	55%
MRC 2–3	4/20	20%
MRC 0–1	5/20	25%
Proximal UL		
MRC 4–5	7/20	35%
MRC 2–3	8/20	40%
MRC 0–1	5/20	25%
Proximal LL		
MRC 4–5	7/20	35%
MRC 2–3	7/20	35%
MRC 0–1	6/20	30%
Assymetry	2/21	10%
Non-ambulant	7/18	38%
Axial weakness	18/19	95%
Facial weakness	9/20	40%
Ptosis	6/20	25%
Respiratory involvement	15/19	79%
**Histopathology**		
Fiber size variation	13/13	100%
Internal nuclei	11/13	85%
Autophagic vacuoles	4/13	31%
Myofibrillar disorganisation	4/13	31%
p62 positive accumulations	5/5	100%
TDP43 positive accumulations	2/2	100%
TUBA4A positive accumulations	6/6	100%
Nemaline bodies	4/13	31%

Each feature is shown as x/y under “Total”, where ‘x’ is the number of individuals exhibiting the feature, and ‘y’ is the number of individuals for whom data were available in that category. The corresponding percentage is calculated from these values. Denominators vary due to missing data.

a Inheritance for Patient 14 is suspected paternal but cannot be determined.

**Table 2:** Histopathological features observed in individuals from our myo-tubulinopathy cohort

	Patient 5	Patient 8	Patient 9	Patient 11	Patient 12	Patient 13	Patient 15	Patient 16	Patient 17	Patient 18	Patient 24	Patient 25	Patient 26
Variation in Fiber size	Y	Y	Y	Y	Y	Y	Y	Y	Y	Y	Y	Y	Y
Internal nucleation	Y	Y	Y	Y	Y	N	Y	Y	Y	Y	N	Y	Y
Type 1 Fiber predominance	N	N	Y	N	Y	Y	N	N	N	Y	N	N	Y
Endomysial Fibrosis	Y	Y	Y	Y	Y	N	Y	N	Y	Y	N	Y	N
Autophagic Vacuoles	N	Y	Y	N	N	N	N	N	N	N	Y	N	Y
Nemaline bodies^a^	N	N	N	N	Y	N	N	N	N	N	N	Y	Y
Spheroid bodies	Y	N	Y	N	N	N	N	N	Y	Y	N	Y	N
Cores	Y	N	N	N	N	N	Y	N	N	N	Y	N	N
Myofibrillar disorganisation	N	Y	N	N	N	N	Y	N	N	N	Y	N	Y
TDP-43 positive accumulations	NA	NA	NA	NA	Y	NA	NA	NA	NA	NA	Y	NA	NA
p62 positive accumulations	NA	Y	Y	Y	Y	NA	NA	NA	NA	NA	Y	NA	NA
TUBA4A positive accumulations	Y	Y	Y	Y	Y	NA	NA	NA	Y	NA	NA	NA	NA

Detailed histopathological data were available for n = 13 individuals. Features are recorded as Y (Yes) or N (No) for each individual. NA= Not accessed.

a Affected father of Patient 2 also showed nemaline bodies on his muscle biopsy. Total number of cases with nemaline bodies = 4

**Table 3:** Assessment of the 13 *TUBA4A* variants observed in our myo-tubulinopathy cohort

TUBA4A variants NM_006000.3	Population frequency gnomAD4.1.0	Family ID; Proband IDs	Age of onset and Sex	Clinical evidence				Revised Classification
				Phenotype	Pattern of Inheritance	genotype/segregation	*n*[N]	Assertion
c.33G>C(p.Gln11His)	0	Family 17; Patient 24	12 years; male	Multisystem proteinopathy	*De novo*	Heterozygous	1[1]	LP
c.34G>A(p.Ala12Thr)	0	Family 19; Patient 26	60 years; male	Multisystem proteinopathy	Autosomal recessive	Homozygous	1[1]	VUS
c.536C>T(p.Thr179Ile)^a^	0	Family 1; Patient 2	At birth; male	Myopathy	Autosomal dominant	Heterozygous	4[1]^b^	P
c.536C>T(p.Thr179Ile)^a^	0	Family 2; Patient 5	42 years; male	Myopathy	Sporadic	Heterozygous	1[1]	P
c.679C>T(p.Leu227Phe)^a^	0	Family 3; Patient 6	14 months; female	Myopathy	Sporadic	Heterozygous	1[1]	P
c.679C>T(p.Leu227Phe)^a^	0	Family 4; Patient 7	At birth; male	Myopathy	*De novo*	Heterozygous	1[1]	P
c.722C>T(p.Ser241Phe)	0	Family 15; Patient 21	12 years; male	Myopathy	Autosomal recessive	Homozygous	1[1]	P
c.760G>A(p.Glu254Lys)	0	Family 5; Patient 8	At birth; male	Myopathy	*De novo*	Heterozygous	1[1]	P
c.760G>C(p.Glu254Gln)	0	Family 6; Patient 9	At birth; male	Myopathy	Sporadic	Heterozygous	1[1]	LP
c.761A>G(p.Glu254Gly)	0	Family 7; Patient 10	At birth; male	Myopathy	*De novo*	Heterozygous	1[1]	P
c.761A>C(p.Glu254Ala)	0	Family 8; Patient 11	At birth; female	Myopathy	Sporadic	Heterozygous	1[1]	P
c.849C>G(p.His283Gln)	0	Family 9; Patient 12	3 years; female	Myopathy	Sporadic	Heterozygous	1[1]	LP
c.850G>A(p.Glu284Lys)^a^	0	Family 10; Patient 13 Family 11; Patient 14	Family 10; Patient 13 = 46 years; female Family 11; Patient 14 = 7 years; female	Myopathy	Sporadic	Heterozygous	2[2]^c^	LP
c.850G>A(p.Glu284Lys)^a^	0	Family 12; Patient 15 Family 13; Patient16A Family 14; Patient 17	Family 12; Patient 15 = 19 years; male Family 13; Patient 16A = 35 years; female Family 14; Patient 17 = 7 years; male	Myopathy	Autosomal dominant	Heterozygous	11[3]^d^	LP
c.1049G>T(p.Gly350Val)	0	Family 18; Patient 25	25 years; female	Myopathy	*De novo*	Heterozygous	1[1]	P
c.1061G>A(p.Gly354Asp)	0	Family 16; Patient 22	first year; female	Myopathy	Autosomal recessive	Homozygous	2[1]^e^	P

n=number of confirmed affected individuals (31), N=number of families (19). P=Pathogenic, LP=Likely pathogenic, VUS=Variant of uncertain significance.

a Indicates recurring variants.

b Patient 2 has two siblings and an affected father harbouring the same heterozygous p.Thr179Ile variant inherited with an Autosomal dominant myopathy.

c Patient 13 and Patient 14 are sporadic cases from two different families, harbouring the p.Glu284Lys variant.

d Patient 15 and six other affected relatives from one family, Patient 16A and Patient 16B from a second family, and Patient 17 and Patient 18 from a third family all harbour the same heterozygous p.Glu284Lys variant associated with an Autosomal dominant myopathy.

e Patient 22 and Patient 23 are sisters harbouring the same homozygous p.Gly354Asp variant inherited with an Autosomal recessive myopathy.

## Data Availability

All *in silico* datasets generated in this study are provided in the Supplementary Data 1. All imaging data directly supporting the findings are included in the manuscript or supplementary figures. Additional imaging files and biological materials used in this study are available from the corresponding authors upon reasonable request (or accessible through commercial providers, where applicable). Sensitive patient sequencing data (sr-ES/sr-GS) have been deposited in *seqr* and the RD-Connect GPAP platform, with controlled access in place to protect participant privacy. All code used for *in silico*, mechanistic, and correlation analyses are publicly available at: https://github.com/RAVING-Informatics/TUBA4A
