# Supplementary material for: Missense variants in TUBA4A cause myo-tubulinopathies
Source: Brain. Author manuscript; Available in PMC 2026 Aug 29. (PMC13524141; doi:10.1093/brain/awag059)
Supplement: Supplementary file 1 [file NIHMS2203371-supplement-Supplementary_Author_Info.pdf]

# Author contributions

Conceptualisation of the study: MJ, GR

Project administration: MJ, GR

Funding acquisition: MJ, GR, NGL

Patient samples and clinical/genomic data collection: All clinicians and molecular geneticists

Data analysis and curation: MJ, CF, JMP, MMO, RVB, YS, SK, MA, EYZ, HRL, CCB, SAS, EAE, LMK, PBK, IOO, BM, LP, MOL, CAT, AODL, FCL, ADA, AC, MT, AP, CVG, GS, MLMN, MC, CAG, ZMV, AHB, VM, GK, AK, AT, NSP, JV, CGC, EZ, CMM, MS, GT, VS, BU, IN, GR

Methodology: MJ, YS, MMO, RVB, JMP, GR

Visualisation: MJ, YS, MMO, JMP, KSS, IAC, DA, SK, CMM, GS, NSP, JV

Writing the original draft: MJ, YS, MMO, JMP, GR

Review and editing of the manuscript: All authors
