# Supplementary material for: Missense variants in TUBA4A cause myo-tubulinopathies
Source: Brain. Author manuscript; Available in PMC 2026 Aug 29. (PMC13524141; doi:10.1093/brain/awag059)
Supplement: Supplementary file 2 [file NIHMS2203371-supplement-Supplementary_Data.pdf]

Johari et al. Missense variants in *TUBA4A* cause myo-tubulinopathies

Supplementary data 1

PART 1: Clinical summary of each patient/family accompanied by brief details of genetic analyses and relevant ethical permissions. Probands are marked with a black arrow and individuals from who DNA sample was available and genotypes are marked with '*'. Individual families are annotated with their proband IDs and corresponding TUBA4A substitutions. Genotypes shown beneath individuals denote the *TUBA4A* variant (gDNA)

1. Family 1 - Proband = Pat.2, Male
   TUBA4A variant details: chr2-219251163-G-A (hg38)
   c.536C>T(p.Thr179Ile)

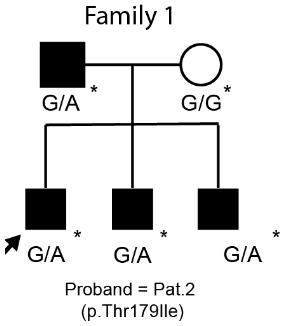

Country: Australia, Contact(s): Clair Habib, Michael Fahey

Clinical summary: The proband is the eldest male sibling of three affected brothers. Their father was affected from the same disease as well. Father was diagnosed at the age of 23 yrs. Muscle biopsy was only available from the father and showed Nemaline bodies on Gomori trichome. Proband was first diagnosed at two years and presented with gross motor delay, waddling gait and proximal weakness. Last examination was done at 17 years of age when he showed mild proximal and distal UL/LL weakness. He has been using a wheelchair since he was five years of age. He has mild bifacial weakness and respiratory issues.

Genetic analysis: Targeted gene panel analysis for Nemaline myopathy was negative. Whole genome analysis was performed on father, unaffected mother and three affected sons. Data was analysed using *seqr* (CPG).

Inheritance: Autosomal dominant

Variant segregation: Yes

Ethical permission: Human Research Ethics Committee, University of Western Australia

2. Family 2 - Proband = Pat.5, Male
   TUBA4A variant details: chr2-219251163-G-A (hg38)
   c.536C>T(p.Thr179Ile)

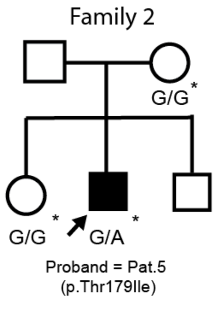

Country: Japan, Contact(s): Yoshihiko Saito, Ichizo Nishino, Joe Nemoto

Clinical summary: The patient was the only affected person in the family; his parents and elder sister and younger brother were not affected. Muscle biopsy was performed only on the proband and showed many fibers with multiminicore on NADH-TR staining. The proband developed muscle weakness in both lower extremities at age 42 years, with difficulty standing and a waddling gait. His last examination was at age 47 and showed mild proximal UL/LL weakness.

Genetic analysis: Trio whole genome analysis was performed on proband and unaffected mother and elder sister. Data was analysed using custom in house pipeline in Department of Neuromuscular Research, National Institute of Neuroscience, National Center of Neurology and Psychiatry, Kodaira, Tokyo, Japan.

Inheritance: Sporadic case

Variant segregation: additional family members not available.

Ethical permission: Ethics Committee, National Center of Neurology and Psychiatry, Tokyo, Japan

3. Family 3 - Proband = Pat.6, Female
TUBA4A variant details: chr2-219251020-G-A (hg38)
c.679C>T(p.Leu227Phe)

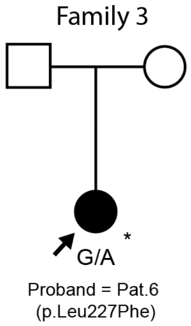

Country: Serbia, Contact (s): Vedrana Milic Rasic, Gordana Kovacevic, Ana Kosac

Clinical summary: early onset (<2 y, about 14 month), some motor improvement during the course of the disease, normal cognitive profile, axial involvement with "rigid spine syndrome", mild scoliosis, distal hypotrophy and weakness of upper and lower extremities, ophthalmoparesis, mild facial weakness, gothic palate, two episodes with swallowing problems.

Diagnostic procedures: Mild hyper-CK, myopathic ENMG, muscle biopsy: vacuolar myopathy with anti-calsequestrin Ab

Genetic analysis: Whole exome analysis was performed through MYO-SEQ. Data was analysed using *RD-Connect* (Solve-RD).

Inheritance: Sporadic case

Variant segregation: additional family members not available.

Ethical permission: Clinic for Neurology and Psychiatry for Children and Youth, Belgrade, Serbia

4. Family 4 - Proband = Pat.7, Male
   TUBA4A variant details: chr2-219251020-G-A (hg38)
   c.679C>T(p.Leu227Phe)

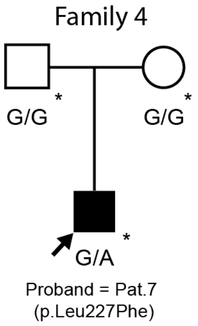

Country: Russia, Contact (s): Sergei Kurbatov

Clinical summary: Prenatal period was normal. The patient had congenital severe hypotonia, weakness, Gowers sign, waddling gait, a long period of drop head and ophthalmoplegia. He has been holding his head in a standing position since 1 y.o, sitting since 1 y 1 month, walking with supported since 2y.o. Every day he improves his motor skills - mild Gowers sign and has been walking unsupported since 4 years old. Last examination was done at 5 years of age when his showed moderate proximal and distal UL/LL weakness, severe weakness flexing neck muscles. He has dry skin since 4. Patient showed signs of fatigability and decrement on RNS. Consequently, the patient was prescribed Salbutamol 0.1 mg/kg/d, and if no improvement - up to 0.3 mg/kg/d for 2-3 months. At 2,5 years weighing 16 kg the patient has been taking salbutamol 5 mg twice daily for 10 days without any improvement in muscle functions.

RNS was performed again at the age of 3,5 years and showed not so convincing decrement up to 17%.

Genetic analysis: Trio whole genome analysis was performed on proband and unaffected mother and father. Data was analysed using custom in house pipeline in Russia and Folkhälsan Research Center, Helsinki, Finland.

Inheritance: heterozygous *de novo*

Variant segregation: Yes

Ethical permission: HUS Ethics Committee (HUS/16896/2022)

5. Family 5 - Proband = Pat.8, Male
   TUBA4A variant details: chr2-219250939-C-T (hg38)
   c.760G>A(p.Glu254Lys)

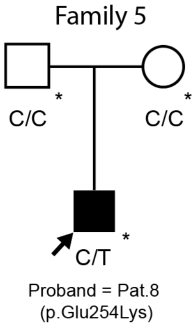

Country: Poland (White, Non-hispanic), Contact(s): Casie Genetti, Alan Beggs, Hirofumi Komaki

Clinical summary: This 11-year-old boy (BOS1139-1) was born full term following a pregnancy complicated by polyhydramnios. At birth APGARs were 9, but hypotonia and floppiness was first appreciated at 3 months of age. At age 2.5 he exhibited typical/excellent cognitive function for age but was unable to sit independently and needed back support while sitting. He has significant upper extremity weakness and could grasp but not reach. Deep tendon reflexes were absent, he remained hypotonic with poor head control, was unable to lift his legs while laying down and unable to roll or rise to sitting on his own. By age 11 he is cognitively intact but has severe proximal and distal weakness of all limbs, severe facial weakness and ptosis, and respiratory involvement requiring ventilatory support since age 7. Laboratory testing is remarkable for mildly elevated serum CK in the 200's, and with myopathic findings on EMG. CPK 04/21/2010: 235, 03/31/2009: 280

Genetic analysis: Trio whole exome sequencing (WES) was performed on the proband and his parents by the Broad Institute Center for Mendelian Genomics on their standard research WES pipeline and variants were analysed on a seqr platform. Biological paternity and maternity were confirmed and a single heterozygous de novo missense variant in TUBA4A was identified. Genomic PCR and Sanger sequencing of the region was employed to orthogonally confirm the variant. No additional likely genetic causes of myopathy were found upon review of the trio WES data.

The proband's genotype is

NC_000002.12:g.[219250939C>T];[=]

NM_006000.3:c.[760G>A];[=]

NP_005991.1:p.[(Glu254Lys)];[=]

Inheritance: *de novo*

Variant segregation: Yes

Ethical permission: Institutional Review Board of Boston Children's Hospital.

6. Family 6 - Proband = Pat.9, Male
TUBA4A variant details: chr2-219250939-C-G (hg38)
c.760G>C(p.Glu254Gln)

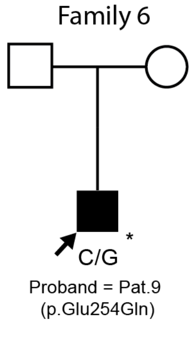

Country: Japan, Contact(s): Yoshihiko Saito, Ichizo Nishino, Takashi Okabe

Clinical summary: The patient was the only affected person in the family; his parents were not affected. Muscle biopsy was performed only on the proband and showed markedly disorganized intermyofibrillar networks on NADH-TR staining, indicating immaturity. Probands was first diagnosed at birth with floppy infant, and facial and generalised muscle weakness. His last examination was at 4 months and showed severe proximal and distal UL/LL weakness.

Genetic analysis: Whole exome analysis was performed only on proband. Data was analysed using custom in house pipeline in Department of Neuromuscular Research, National Institute of Neuroscience, National Center of Neurology and Psychiatry, Kodaira, Tokyo, Japan.

Inheritance: Sporadic case

Variant segregation: additional family members not available.

Ethical permission: Ethics Committee, National Center of Neurology and Psychiatry, Tokyo, Japan

7. Family 7 - Proband = Pat.10, Male
TUBA4A variant details: chr2-219250938-T-C (hg38)
c.761A>G(p.Glu254Gly)

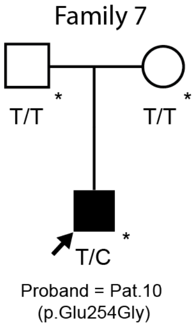

Country: Italy, Contact(s): Adele D'Amico, Marco Tartaglia

Clinical summary: Third-born child. Delivered at 39 weeks by elective Caesarean section. Birth weight: 3090 g. At birth, the infant presented with respiratory distress requiring oxygen therapy for 5 days. He consistently exhibited weak sucking and was never able to swallow; feeding was administered via gavage. Generalized hypotonia was noted from birth, with more pronounced involvement of the upper limbs. Metabolic screening and brain MRI were both unremarkable. Electromyography (EMG) revealed polyphasic motor unit potentials and a myopathic recruitment pattern. No fibrillation activity was detected. Creatine phosphokinase (CPK) levels were within normal limits.

Neurological examination at 40 days of life showed normal attention and visual tracking. The infant had a hypotonic facial expression with mild micrognathia. Spontaneous motor activity was markedly reduced, limited primarily to ocular movements. He exhibited a frog-leg posture and generalized muscle weakness. Deep tendon reflexes were absent. Cryptorchidism was noted. The respiratory pattern was appropriate for age; however, chest X-ray revealed an early 'bell-shaped' thoracic deformity, suggestive of intercostal muscle involvement.

Muscle biopsy showed evidence of severe myopathic changes, with increased intramuscular glycogen content.

The clinical course was rapidly progressive, and the infant passed away at 9 months of age due to severe respiratory failure.

Genetic analysis: exome sequencing

Inheritance: *de novo*

Variant segregation: Yes, confirmed by Sanger sequencing of proband and parents.

Ethical permission: Ospedale Pediatrico Bambino Gesù Ethical Committee (1702_OPBG_2018)

8. Family 8 - Proband = Pat.11, Female
TUBA4A variant details: chr2-219250938-T-G (hg38)
c.761A>C(p.Glu254Ala)

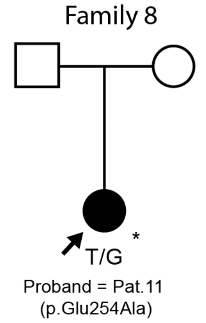

Country: Japan, Contact(s): Yoshihiko Saito, Ichizo Nishino, Yasuhiro Kimoto

Clinical summary: The patient was the only affected person in the family; his parents were not affected. Muscle biopsy was performed only on the proband and showed many fibers with peripheral halo on NADH-TR staining, indicating immaturity. Proband was first diagnosed at birth with floppy infant, and facial and generalized muscle weakness. Her last examination was at 5 months and showed severe proximal and distal UL/LL weakness.

Genetic analysis: Whole exome analysis was performed only on proband. Data was analysed using custom in house pipeline in Department of Neuromuscular Research, National Institute of Neuroscience, National Center of Neurology and Psychiatry, Kodaira, Tokyo, Japan.

Inheritance: Sporadic case

Variant segregation: additional family members not available.

Ethical permission: Ethics Committee, National Center of Neurology and Psychiatry, Tokyo, Japan

9. Family 9 - Proband = Pat.12, Female
TUBA4A variant details: chr2-219250850-G-C (hg38)
c.849C>G(p.His283Gln)

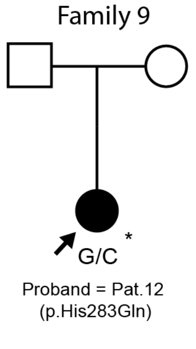

Country: Japan, Contact(s): Yoshihiko Saito, Ichizo Nishino, Takeshi Tokito

Clinical summary: The patient was the only affected person in the family; her parents were not affected. Muscle biopsy was performed only on the proband and showed many fibers with nemaline bodies on mGT staining. The proband developed muscle weakness in both lower extremities at childhood, with difficulty running and a waddling gait. Her last examination was at age 10 and showed mild proximal UL/LL weakness.

Genetic analysis: Whole exome analysis was performed only on proband. Data was analysed using custom in house pipeline in Department of Neuromuscular Research, National Institute of Neuroscience, National Center of Neurology and Psychiatry, Kodaira, Tokyo, Japan.

Inheritance: Sporadic case

Variant segregation: additional family members not available.

Ethical permission: Ethics Committee, National Center of Neurology and Psychiatry, Tokyo, Japan

10. Family 10 - Proband = Pat.13, Female
TUBA4A variant details: chr2-219250849-C-T (hg38)
c.850G>A(p.Glu284Lys)

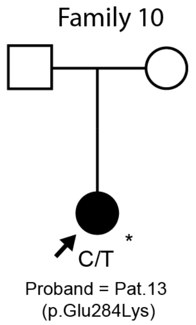

Country: France, Contact(s): Guilhem Sole, Mireille Cossee

Clinical summary: The proband presented with axial muscular weakness associated with scapular winging at age 44. There was no familial history. At last evaluation when she was 60 axial, proximal lower and upper limbs were weak. Facial and distal muscle strength was normal. Cardiac evaluation was normal. Forced vital capacity was low at 70%. Serum CK level was high at 602. Myography showed a mixed pattern of neurogenic and myogenic abnormalities. Muscle biopsy showed a wide variation in myofibre size range with numerous round in shape atrophic myofibres and numerous lobulated myofibres. Muscle MRI showed Adipose involution with amyotrophy of paravertebral, vastus lateralis, semimembranosus, biceps femoris, semitendinosus medial gastrocnemius and soleus muscles.

Genetic analysis: Exome sequencing

Inheritance: Sporadic case

Variant segregation: additional family members not available.

Ethical permission: National Data Protection Commission (Commission Nationale de l'Informatique et des Libertés), #918347

11. Family 11 - Proband = Pat.14, Female
TUBA4A variant details: chr2-219250849-C-T (hg38)
c.850G>A(p.Glu284Lys)

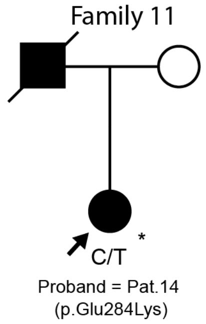

Country: USA, Contact(s): Christina Austin-Tse, Lynn Pais

Clinical summary: Proband's symptoms emerged at age 7 with difficulty running and easy fatigability. She experienced slowly progressive shoulder and pelvic weakness and passed away at age 57 from reported lung issues. Muscle biopsy at age 18 confirmed an unspecified muscle disease. Her father had a history of muscle weakness and difficulty ambulating.

Genetic analysis: Proband-only whole genome sequencing was performed.

Inheritance: Unknown, possibly paternal

Variant segregation: additional family members not available.

Ethical permission: Mass General Brigham IRB (Protocol# 2016P001422)

12. Family 12 - Proband = Pat.15, Male
TUBA4A variant details: chr2-219250849-C-T (hg38)
c.850G>A(p.Glu284Lys)

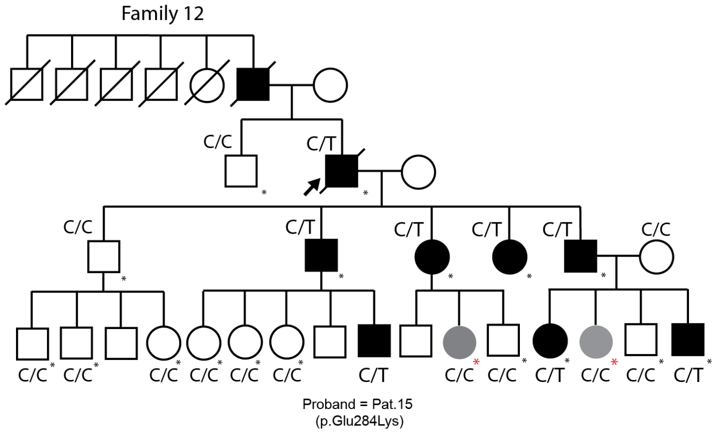

Country: USA, Contact(s): Elicia A. Estrella, Peter Kang

Clinical summary: Proband was a man in his 70's when he passed. He had onset of disease around age 19 with a slow progression of muscle weakness proximal>distal. He lost ambulation in his mid 40's but was still able to bear weight on his legs to assist with transfer. He had elevated serum CK levels, a myopathic EMG, and a dystrophic muscle biopsy. His initial symptoms were kyphoscoliosis and abnormal posture, which has been seen in the next two generations, indicating a likely autosomal dominant inheritance pattern.

Genetic analysis: Exome sequencing was performed on the proband, daughter-in law (unaffected), son (affected), daughter (affected), and son (unaffected). Sanger sequencing was performed to confirm the WES results, and to determine segregation patterns in additional family members. PCR and Sanger sequencing was performed using the following primers: TUBA4A-F 5' TCA GGT CGA ACT TGT GGT CCA G 3' and TUBA4A-R 5' CTT TCT CGT TCT TGC CTT TCA GTC 3'. Sanger sequencing was performed by Eurofins Genomics.

Inheritance: Autosomal Dominant

Variant segregation: Yes, in all individuals with phenotype assessments pending on two individuals. In generations 1 and 2, the variant segregates with affected status and with CK values. In all 3 generations, the variant segregates with CK values (total of 10 individuals). In generation 3, there are two individuals with the variant who have elevated CKs and are reported to be affected. There are 2 individuals in generation 3 for whom phenotype assessments are pending. A CK is pending for one of these individuals, and a reassessment of the other individual's phenotype status is ongoing.

Ethical permission: Subjects were enrolled under the auspices of a protocol approved by the institutional review board (IRB) of Boston Children's Hospital.

13. Family 13, Proband = Pat.16A, Female and affected daughter Pat.16B
    TUBA4A variant details: chr2-219250849-C-T (hg38)
    c.850G>A(p.Glu284Lys)

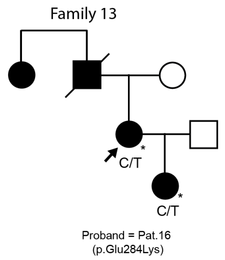

Country: Denmark, Contact(s): John Vissing

Clinical summary: The proband is now 70 years old. Her deceased father had the same disease. Father's sister is reportedly affected too. She started developing symptoms around age 40 years, when she got difficulties getting up from the floor. Gradually lost ambulation and wheelchair bound the last 10 years. Now also proximal weakness of arms making eating independently difficult. Normal echocardiography and FVC of 60% of normal. Non-invasive ventilation at night from age 70 years. Her now 29-year-old daughter carries the same variant and is asymptomatic but has elevated CK (466), fatty replacement of semitendinosus and medial gastrocnemius on MRI and mild proximal lower limb weakness.

Genetic analysis: WES done through MYO SEQ

Inheritance: Autosomal Dominant

Variant segregation: Limited as additional family members not available.

Ethical permission: The Committee on Health Research Ethics of the Capital Region of Denmark (approval number H-19086169)

14. Family 14, Proband = Pat.17, Male and affected mother Pat.18
TUBA4A variant details: chr2-219250849-C-T (hg38)
c.850G>A(p.Glu284Lys)

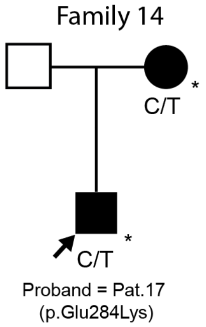

Country: Japan, Contact(s): Yoshihiko Saito, Ichizo Nishino, Masaki Nakanowatari, Yasushi Oya

Clinical summary: The proband and her son are affected. They were diagnosed at the age of 31 yrs and 26yrs. Muscle biopsy samples were available from the mother and son and showed scattered myofibres with internalised nuclei and spheroid bodies on Gomori trichome. Mother and son were first diagnosed at 28 years and 7 years and presented with proximal weakness. Last examination was done at 31 and 26 years of age when they showed mild proximal and distal UL/LL weakness.

Genetic analysis: Whole genome analysis was performed on proband and son. Data was analysed using custom in house pipeline in Department of Neuromuscular Research, National Institute of Neuroscience, National Center of Neurology and Psychiatry, Kodaira, Tokyo, Japan.

Inheritance: Autosomal Dominant

Variant segregation: Yes

Ethical permission: Ethics Committee, National Center of Neurology and Psychiatry, Tokyo, Japan

15. Family 15 - Proband = Pat 21
TUBA4A variant details: chr2-219250977-G-A (hg38)
c.722C>T(p.Ser241Phe)

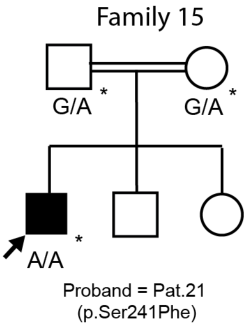

Country: Netherlands, Contact(s): Nicol Voermans, Thatjana Gardeitchik

Clinical summary: Pat.21 had first onset of symptoms at age 12 years old and was last examined at age 19 years where he showed severe distal upper and lower limb weakness along with severe proximal upper and lower limb weakness. CK was 2,794 IU/l. There was no family history of a neurological disease, and both parents (consanguineous) did not have neuromuscular symptoms and neurological examination in both was normal. EMG of the patient was abnormal and RNS of ulnar nerve showed decrement.

Rep Nerve Stim

Anatomy / Train	Time	Rate Hz	Ampl. 1-2 mV	Ampl I-IV %	Area mVms	Area I-IV %
**R Abductor digiti minimi**						
**Start 3 Hz**	0:00:00	3	5,9	**-24,5**	12,9	-28,5
**3 Hz na 30 s aansp**	0:00:50	3	5,5	-8,2	12,5	-11,3
**R Trapezius (upper)**						
**Start 3 Hz**	0:00:00	3	7,0	-5,9	20,8	-10,9
**3 Hz na 30 s aansp**	0:00:41	3	8,0	3,3	21,3	0,9
**R Nasalis**						
**Start 3 Hz**	0:00:00	3	1,3	-3,7	3,0	-3,4
**3 Hz na 30 s aansp**	0:01:08	3	1,3	16,2	2,9	23,8

RNS of right ADM was 24.5 %. There is no long term follow up since he returned to the regional hospital. Initially, there was a possible effect of pyridostigmine.

Genetic analysis: targeted gene panel (neuromuscular genes) and full exome trio sequencing.

Inheritance: Autosomal Recessive

Variant segregation: Yes (Both parents are heterozygous carriers)

Ethical permission: Medical Review Ethics Committee, Region Arnhem–Nijmegen, Number 2011/188

16. Family 16, Proband = Pat.22, Female and affected sister Pat.23
TUBA4A variant details: chr2-220115360-C-T (hg38)
c.1061G>A(p.Gly354Asp)

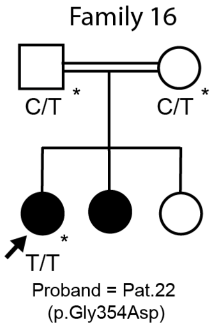

Country: Netherlands, Contact(s): Laura Donker Kaat & Evita Medici

Clinical summary: Patient 22 (currently 15 years old) showed motor delay since infancy. Sitting at age 1 but never able to walk. Normal speech and cognitive development. First clinical examination at age 12 years at our hospital revealed severe scoliosis, reduced lung capacity, generalized symmetrical weakness more severely in legs than arms with contractures in elbows and knees. She was wheelchair bound. There were no swallowing problems. The family history showed an affected sister (pat 23) and a healthy younger sister. Both parents (consanguineous) did not have neuromuscular symptoms and neurological examination in both was normal. Because her sister (pat 23) showed signs of neuromuscular junction disfunction, pyridostigmine was started and showed some improvement in motor functioning.

Her sister (patient 23, currently 14 years old) also showed motor delay since infancy. She could sit at 1 year and walk at 2,5-3 years but never long distances. Normal speech and cognitive development. First clinical examination at age 10 years at our hospital revealed subtle facial weakness and generalized symmetrical weakness proximal and distal MRC 4. She was wheelchair bound and was able to take a few steps. There were swallowing problems and tube feeding was started. Because repetitive nerve stimulation showed decrement response, pyridostigmine was started and a mild, but transient improvement in motor function was

observed. Besides her neuromuscular phenotype, she also suffered from recurrent respiratory infections due to CARLIM2-immunodeficiency.

Genetic analysis: SNP-array, SMN1 gene, targeted gene panel (neuromuscular genes) and full exome trio sequencing.

Inheritance: Autosomal Recessive

Variant segregation: Yes (both parents are heterozygous carriers)

Ethical permission: Medical Review Ethics Committee, Region Arnhem–Nijmegen, Number 2011/188

17. Family 17 - Proband = Pat.24, Male
TUBA4A variant details: chr2-219252201-G-C (hg38)
c.33G>C(p.Gln11His)

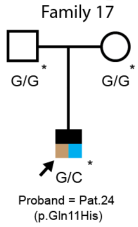

Country: Brazil, Contact(s): Cristiane Araujo Martins Moreno, Clara Gontijo Camelo, Edmar Zanoteli

Clinical summary: first child of nonconsanguineous parents. Motor Milestones normal, but parents noticed frequent falls in the first years. He was never able to jump and had difficulties to run and stand from floor. He developed with exercise intolerance and was diagnosed with myopathy. At 6 years old he started with epileptic seizures treated with more than one drug and learning difficulties. At clinical exam he presented broad forehead, arched eyebrows, numerous eyelashes, apparent hypertelorism, downward-slanting palpebral fissures, bulbous nose, long and subtly defined philtrum, thin upper lip, thick and everted lower lip, ears with thickened and forward-facing helix. He was hypotonic and presented mild proximal weakness and reduced tendon reflexes. Brain MRI showed no abnormalities and CK was mildly elevated (387 – 1.5X elevation). Muscle biopsy presented increased myofibre size variability, nuclear internalisation, myofibrillar disarrangement and the presence of vacuoles., TDP43, p62 and Desmin-positive staining.

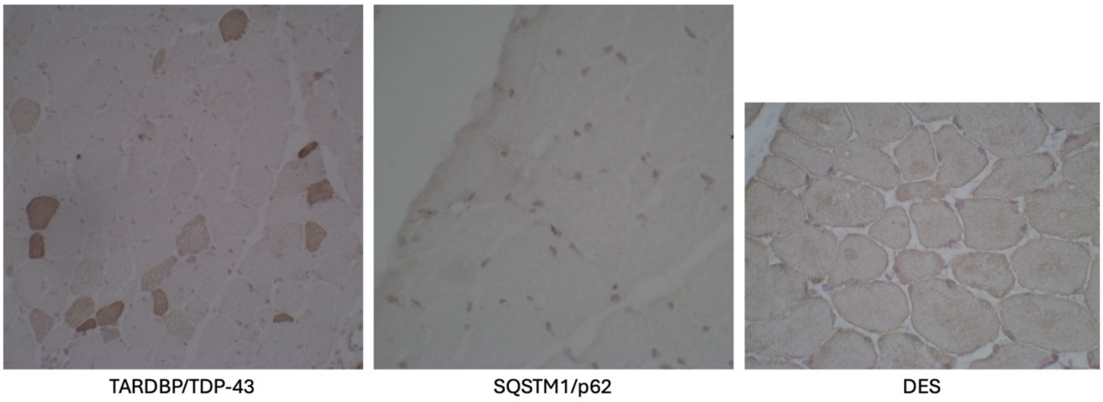

Genetic analysis: WES showed a heterozygous variant at TUBA4A (p.Gln11His)

Inheritance: *de novo*

Variant segregation: Yes, confirmed by Sanger sequencing

Ethical permission: Clinical Hospital of Universidade de São Paulo Ethical Committee.

18. Family 18 - Proband = Pat.25, Female
TUBA4A variant details: chr2-219250650-C-A (hg38)
c.1049G>T(p.Gly350Val)

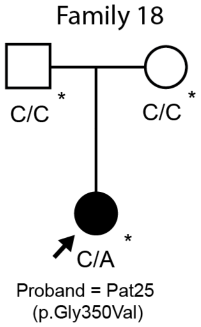

Country: Russia, Contact (s): Sergei Kurbatov

Clinical summary: She was having difficulty passing PE tests since 7. She has never been able to sit on a full foot. Scoliosis was diagnosed from the age of 15. She was suspected of having myasthenia gravis at the age of 31 by neurologist. Attach And RNS with proserpine has been normal, needle EMG - myopathic, CK was 1700. She was suspected scapuloperoneal myopathy, FSHD, SMA 5q, LGMD, no mutation on were found since 31 to 37 y.o. The disorder is slowly progressing, at the age of 31. She has difficulty got up from squatting since 33 (since 36 she couldn't) she has difficulty climbed stairs since 35 (she couldn't without handrails since 37).

Last examination was done at 37 years of age when at height 163 cm, weight 52 kg. No Achilles tendon retraction. Hyperkeratosis pillari. Slight elbow joint contractures and rigidity of the thoracolumbar spine straightened lumbar lordosis. Slight ophthalmoplegia (in extreme lateral leads). Atrophy of distal (thenar, interosseous, feet, forearms, shins) and proximal muscles (shoulder girdle, lower third of thighs). Weakness of neck flexor/extensor muscles up to 2/3 points (she cannot tear her head off the couch lying on her back), tongue muscles up to 3,5-4, flexor/extensor forearm 3,5/3, fingers 5/2, thumb 3/3, biceps 2,5, triceps 4 (no Popeye sign), bringing the outstretched arms together and apart 2,5, deltoids 3, raising the outstretched arms in front of you 2 (no poly-hill sign), abdominal muscles 4 (no Beevor's signs), iliopsoas 3, anterior thigh 3,5, hamstrings 4, flexor/extensor feet and extensor toe 3,5, flexor toe 4. She has fatigue after exercising (but not much). Tendon reflexes are absent. The gait is waddling, it is difficult to get up from a highchair, does not get up from the floor, cannot walk on her heels and a finger, there is no scapular winging. The hands and feet are cold, with slight hyperhidrosis.

Electron microscopy analysis showed glycogen accumulation and presence of nemaline bodies.

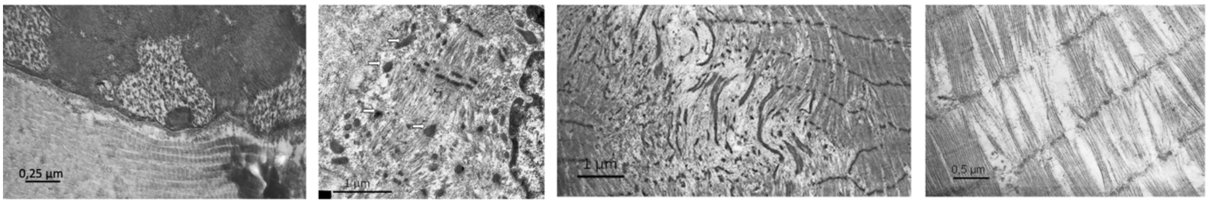

Glycogen accumulation | Nemaline bodies | excessive Z-line, disrupted myofilament | "bamboo-like" fragmentation

The patient showed fatigability and decrements on RNS which prompted a treatment with Salbutamol, 4 mg, if no improvement then up to 8 mg bid for 2-3 months.

At 38 years of age, she has been taking Salbutamol per oris once 5 mg and she got tachycardia and harbingers of a panic attack. Thereafter she has been taking Salbutamol per oris once 2,5 mg and again she got tachycardia and harbingers of a panic attack. The salbutamol treatment was stopped after the last episode.

EMG RNS from ulnar and ocular muscle showed no decrement but clear decrement (up to 22%) from Deltoideus (see it below).

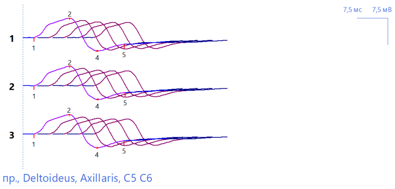

Genetic analysis: Initial Trio-WES analysis in Independent Clinical Bioinformatics Laboratory, 123181 Moscow was performed on proband and unaffected mother and father. Subsequent WGS analysis for proband was performed at Russian Genoned Ltd. Data was analysed at Folkhalsan Research Center (Helsinki, Finland)

Inheritance: *de novo* (hez)

Variant segregation: Yes

Ethical permission: HUS Ethics Committee (HUS/16896/2022)

19. Family 19 - Proband = Pat.26, Male
TUBA4A variant details: chr2-219252200-C-T
c.34G>A(p.Ala12Thr)

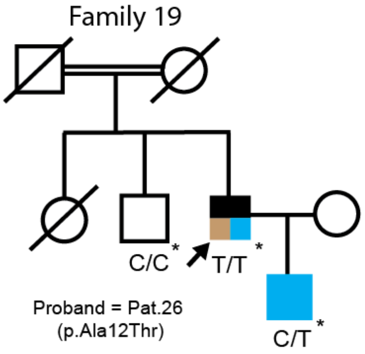

Country: Italy, Contact(s): Giorgio Tasca

Clinical summary: Clinical details of the proband are included in Tasca et al. (PMID: 22806379)

Genetic analysis: Initial targeted gene panel analysis for myofibrillar myopathy was negative. We identified a VUS in *FLNC* (Tasca *et al*. 2012 Muscle and Nerve). Subsequent Myocap gene panel analysis of the proband was negative for known genes (*TUBA4A* was not included as a candidate gene in that version of Myocap) Whole genome analysis was performed at Folkhalsan Research Center (Helsinki, Finland) and data was analysed using custom in house pipelines. Subsequent data analysis showed homozygous TUBA4A p.(Ala12Thr) variant in the proband.

Inheritance: Homozygous recessive

Variant segregation: Unaffected brother does not have the variant, while son with epilepsy has heterozygous TUBA4A p.(Ala12Thr)

Ethical permission: HUS Ethics Committee (HUS/16896/2022)

PART 2: Supplementary Methods and Results

DNA sequencing and analysis

Targeted gene panel sequencing, short read (sr)-exome sequencing (ES) and sr-genome sequencing (GS) were carried out on probands and available family members. Sanger sequencing was performed to confirm the variants identified in the probands and to test for co-segregation of the variants with disease in available family members. Single nucleotide variant (SNV) and indel analyses were done through a) seqr- a web-based platform hosted by the Centre for Population Genomics (CPG; Garvan Institute of Medical Research, Murdoch Children's Research Institute) and the Centre for Mendelian Genomics (CMG; the Broad Institute),[1] b) RD-Connect Genome Phenome Analysis Platform (GPAP),[2] and custom in-house pipelines using a minor allele frequency (MAF) £ 0.0001 in the Genome Aggregation Database (gnomAD) v2.1.1 (hg19),[3] and v3.1.2 (hg38).[4]

TUBA4A variant analysis and interpretation

Variants in *TUBA4A* were annotated on NM_006000.3 (ENST00000248437.9, MANE Select) and NP_005991.1 (protein). Variant frequency reanalysis was performed using the updated gnomAD v4.1.0. In addition to the variants identified in patient cohorts, two likely benign variants (c.541G>A; p.Val181Met and c.125T>C; p.Ile42Thr), each observed in over 100 alleles in gnomAD v4.1.0, were included as population controls for comparative analyses.

Identified variants were assessed using various *in silico* tools per current ACMG/AMP guidelines. The variant assessment was refined using the germline variant classifier VarSome,[5] with a modified pathogenicity interpretation based on the evidence levels available, including those collected during this study.

Genotype-phenotype correlation analysis

Clinical severity was quantified using Medical Research Council (MRC) scores for proximal and distal muscle strength in the upper and lower limbs (5 = normal, 1 = severe weakness). Additional features, including axial weakness, facial weakness, ptosis, and respiratory involvement, were coded as binary variables (0 = absent, 1 = present). Mode of inheritance (autosomal dominant = 1, autosomal recessive = 2, de novo = 3, sporadic = 4), serum CK levels (normal = 0, mildly elevated < 1000 U/L = 1, > 1000 U/L = 2), and TUBA4A variant domains (GTPase = 1, C-terminal = 2, GTP-binding site = 3) were also numerically coded.

Categorical variables were numericalised to enable computation of Pearson's correlation coefficients between clinical and genetic parameters (Supplementary Data 3). Correlation analyses were exploratory and aimed to identify trends rather than to test a formal hypothesis. Consequently, p-values were not emphasised, as the limited cohort size reduces statistical power. Instead, the magnitude and direction of the Pearson correlation coefficient (r) were considered biologically more informative than a binary significance cutoff. Analyses were performed in R (v4.3.1) using the cor.test() function, and results were visualised with ggplot2.

Limitations: Interpretation of genotype–phenotype correlations may be influenced by unequal representation of domains (particularly few C-terminal and GTP-binding variants), age-dependent clinical expression, and inter-centre variability in clinical assessments. Potential genetic modifiers were not accounted for and may contribute to residual confounding. Inheritance pattern was not included in correlation analyses because the categories are nominal and unevenly distributed, precluding meaningful rank-based or parametric correlations.

Code for analysis can be found at: https://github.com/RAVING-Informatics/TUBA4A

Supplementary table 1: Numerical domain classification where: 1 = GTPase domain, 2 = C-terminal domain, 3 = GTP-binding region. Distal UL (DUL), Proximal UL (PUL), Distal LL (DLL), Proximal LL (PLL):Muscle strength assessed using an MRC scale: 5 = normal strength, 1 = severe weakness. Higher values therefore indicate milder phenotype. Binary clinical features (Axial weakness, Facial weakness, Ptosis, Respiratory involvement): 1 = present, 0 = absent, NA = not accessed. Creatine kinase (CK): Ordinal scale: 0 = normal, 1 = mildly elevated, 2 = markedly elevated.

ID	Variant	Domain	Phenotype	Inheritance	DUL	PUL	DLL	PLL	Axial	Facial	Ptosis	Respiratory	CK
Pat2	p.(Thr179Ile)	1	Myopathy	1	4	4	4	4	NA	1	0	1	1
Pat5	p.(Thr179Ile)	1	Myopathy	4	5	4	5	4	1	0	0	0	1
Pat6	p.(Leu227Phe)	1	Myopathy	4	2	5	2	5	1	1	0	0	1
Pat7	p.(Leu227Phe)	1	Myopathy	3	2	2	4	2	1	1	1	NA	0
Pat8	p.(Glu254Lys)	1	Myopathy	3	1	1	1	1	1	1	1	1	0
Pat9	p.(Glu254Gln)	1	Myopathy	4	1	1	1	1	1	1	1	1	1
Pat10	p.(Glu254Gly)	1	Myopathy	3	1	1	1	1	1	1	0	1	1
Pat11	p.(Glu254Ala)	1	Myopathy	4	1	1	1	1	1	1	1	1	1
Pat12	p.(His283Gln)	2	Myopathy	4	4	4	4	4	0	0	0	0	0
Pat13	p.(Glu284Lys)	2	Myopathy	4	5	4	5	4	1	0	0	1	1
Pat15	p.(Glu284Lys)	2	Myopathy	1	4	2	2	1	1	0	0	0	1
Pat16A	p.(Glu284Lys)	2	Myopathy	1	4	2	4	2	1	0	0	1	1
Pat17	p.(Glu284Lys)	2	Myopathy	1	4	4	4	2	1	0	0	1	2
Pat18	p.(Glu284Lys)	2	Myopathy	1	4	2	2	2	1	0	0	1	1
Pat21	p.(Ser241Phe)	1	Myopathy	2	4	2	4	2	1	0	0	1	1
Pat22	p.(Gly354Asp)	2	Myopathy	2	4	2	4	2	1	0	0	1	1
Pat23	p.(Gly354Asp)	2	Myopathy	2	3	3	4	4	1	0	0	1	1
Pat24	p.(Gln11His)	3	Multisystem proteinopathy	3	4	4	4	4	1	0	1	1	1
Pat25	p.(Gly350Val)	2	Myopathy	3	2	2	3	3	1	0	0	1	2
Pat26	p.(Ala12Thr)	3	Multisystem proteinopathy	2	5	3	4	2	1	1	0	1	0

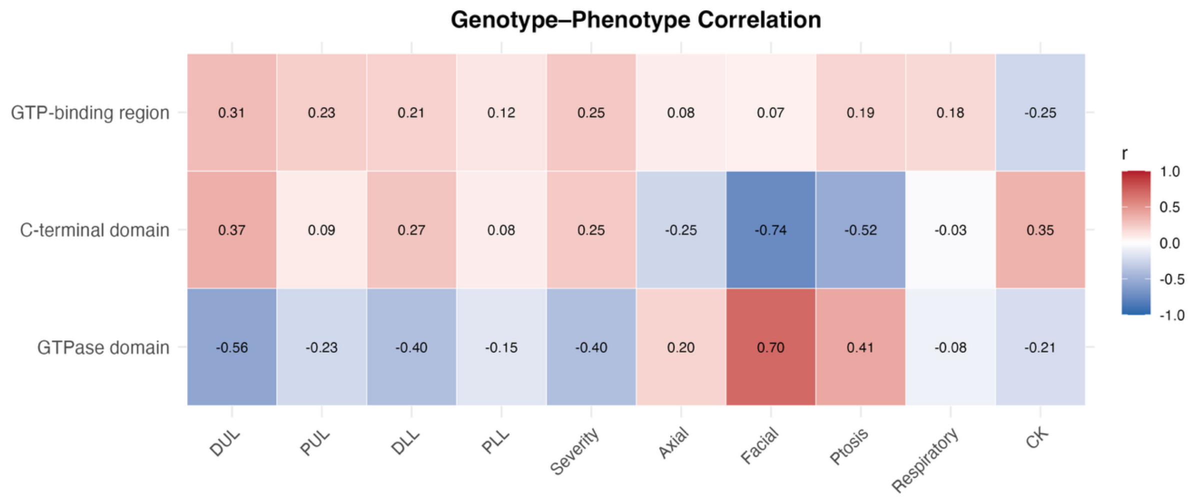

Supplementary Figure 1: Genotype-phenotype correlation in *TUBA4A*-related myo-tubulinopathies

Pearson correlation coefficients (r) are shown for each combination of domain (rows) and clinical variable (columns). Clinical variables include inverted MRC subscores for limb weakness (DUL, PUL, DLL, PLL; 5 = normal, 1 = severe), a composite limb Severity score (mean MRC value, 5-1), binary features (1 = present, 0=absence), and CK elevation (0–2 scale). (Lower MRC and mean MRC value mean more severity). Positive correlations (red) indicate that variants in that domain tend to be associated with higher clinical values. Negative correlations (blue) indicate that variants in that domain tend to be associated with lower clinical values. Effect sizes follow standard conventions: $|r| < 0.3$ = weak, 0.3–0.6 = moderate, > 0.6 = strong. GTPase-domain variants show moderate–strong negative correlations with MRC scores ($r \approx$ –0.40 to –0.56) and Severity (–0.40), indicating more severe limb weakness. They show strong positive correlations with Facial weakness r = +0.70; Ptosis r = +0.41, reflecting that these features, along with severe limb weakness were often seen patients with variants in GTPase domain. C-terminal variants show the opposite pattern: moderate positive correlations with MRC scores and Severity ($r \approx$ 0.25–0.37), indicating milder limb weakness, and strong negative correlations with facial weakness (r = –0.74) and ptosis (–0.52), reflecting near absence of these features in patients where variants are seen in C-terminus (see table). GTP-binding region variants display weak correlations across all features.

***In-silico* modelling of TUBA4A substitutions**

Multiple sequence alignment

To examine the conservation of substituted amino acid positions, we performed multiple sequence alignments of TUBA4A orthologs across different species and human TUBA4A paralogs. TUBA4A ortholog and paralog protein sequences were retrieved from UniProt using their respective accession codes. Alignments were performed using the default MAFFT algorithm in Jalview (v2.11.2).

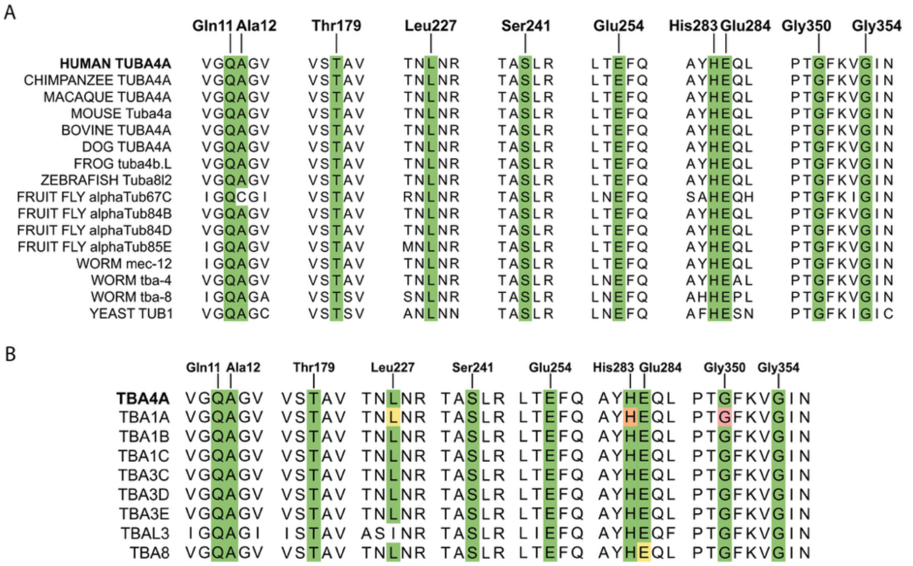

Supplementary Figure 2: A) Conservation of Tubulins across species showed that all identified variants in our myopathy cohort are at highly conserved sites. B) Conservation of identified TUBA4A variant amino acid residues across other human tubulin paralogs. Conserved amino acids are highlighted with a green box. Pathogenic variants (red box), likely pathogenic variants (orange box), and variants of uncertain significance (yellow box) identified in paralogous human tubulins are also shown.

Calculation of free energy changes and TUBA4A aggregation prediction

We used FoldX (v5.0)[6] to investigate the impact of *TUBA4A* variants on protein stability and to show the free energy changes between the wild-type and mutant proteins. Free energy change predictions were only performed on missense substitutions within high confidence regions (pLDDT > 90) of the AlphaFold2-predicted protein structure.[7] The AlphaFold2-derived wild-type TUBA4A protein structure, based on NM_006000.3, was energy-minimised and prepared for mutagenesis with the 'RepairPDB' and 'BuildModel' commands in FoldX, respectively. Subsequent free energy calculations were performed with the 'Stability' command, and differences in free energy were determined by subtracting the free energy value of the wild-type protein monomer from that of the mutant protein (ΔG_{MUT} - ΔG_{WT}). Calculations were replicated 10 times with default parameters for each substitution. Substitutions were classified as having a destabilising effect on protein folding if the free energy difference ($\Delta\Delta G$) was >1.6 kcal/mol.[6,8,9] Furthermore, we calculated the significance of FoldX stability predictions per-variant by comparing the per-variant values against a conservative destabilisation threshold of 1.6 kcal/mol using a bootstrapping approach. Analyses and plotting were performed in RStudio (v2024.12.1+563) R (v4.4.3).

Aggrescan 4D was used to examine the aggregation propensity of wild-type and mutant TUBA4A.[10,11] Since there is no TUBA4A 3D protein structure derived from X-ray diffraction or solution NMR, Aggrescan utilises

modelling approaches in PDB/mmCIF format to predict the aggregation propensities of TUBA4A. The A4D Dynamic mode was used, which integrates the CABS-flex tool to model protein flexibility and incorporate this information into the assessment of aggregation properties.[12] In this approach, the simulation begins with a FoldX-optimised input structure as the starting point.[6] Aggregation propensity scores were calculated for all 448 amino acids of TUBA4A across both wild-type and mutant proteins. The normalised average aggregation propensity score of each TUBA4A mutant protein was calculated relative to wild-type, using the Aggrescan4D-derived scores. Analyses and plotting were performed in RStudio (v2023.06.1) R (v4.3.3).

<u>TUBA4A structural modelling and *in silico* mutagenesis</u>

The predicted wild-type TUBA4A protein structure, based on NM_006000.3/NP_005991.1, was retrieved from the AlphaFold Protein Structure Database[13] using its UniProt accession code (P68366). Three-dimensional protein structures were visualised using PyMol (v. 2.5.2). To examine the effects of variants on three-dimensional protein structure, *in silico* mutagenesis for identified missense substitutions was performed through the PyMol 'mutagenesis' function.

Inclusion of benign variants: To contextualise the molecular impact of pathogenic and VUS substitutions, two likely benign TUBA4A variants, c.541G>A (p.Val181Met) and c.125T>C (p.Ile42Thr), were incorporated as internal controls in all in silico analyses. These variants were selected based on their high population frequency (>100 alleles in gnomAD v4.1.0) and absence of reported neuromuscular or neurodegenerative phenotypes. Both variants were included in all subsequent FoldX stability predictions, Aggrescan4D aggregation analyses, and PyMOL structural modelling, following identical workflows as used for the pathogenic candidates. Structural inspection of these two benign variants revealed minor alterations to polar contacts without steric clashes or perturbation of local packing.

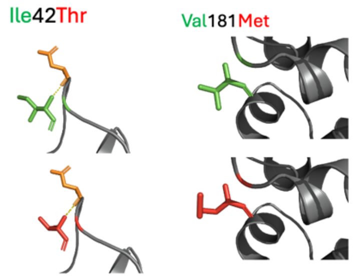

Supplementary Fig 3: The effect of benign variants on three-dimensional TUBA4A structure. Wild-type residues are coloured green and mutant residues are coloured red.

To examine whether the variants affect any binding sites, an experimentally validated model of mouse TUBA4A-TUBB2A with kinesin (PDB: 8IXF)[14] was visualised, and images were exported using Mol*.[15]

***In vitro* over-expression of *TUBA4A* variants in COS1 cells**

Wild-type human *TUBA4A* ORF was cloned into pcDNA3 to produce an N-terminally HA-tagged construct. The observed missense variants were introduced to the construct by site-directed mutagenesis. Wild-type and mutant *TUBA4A* were overexpressed in COS1 cells to study the localisation of TUBA4A and microtubule morphology. COS1 cells were cultured in Dulbecco's Modified Eagle Medium (DMEM) supplemented with 10% fetal calf

serum (FCS). Cells were grown on glass coverslips and, at approximately 60% confluence, transfected with Lipofectamine 2000 (Invitrogen) according to the manufacturer's procedure. Cells were transfected with either WT or *TUBA4A*-myopathy variants: p.(Thr179Ile), p.(Leu227Phe), p.(Ser241Phe), p.(Glu254Ala), p.(Glu254Gln), p.(Glu254Lys), p.(Glu254Gly), p.(Gly354Asp). Additionally, we also transfected cells with previously identified TUBA4A ALS-causing substitution p.(Arg105Cys) and p.(Arg320Cys).[16] Upon 48 hours of incubation, the cells were fixed with ice-cold methanol for 10 minutes at room temperature (RT) and blocked with 2% bovine serum albumin (BSA) in PBS, followed by 1-hour incubation with the primary anti-HA antibody (mouse monoclonal, Sigma-Aldrich, Zwijndrecht, Netherlands), a PBS wash and another 1 hr incubation with the secondary anti-mouse Alexa Fluor-488 antibody (ThermoFisher Scientific Waltham, USA). The coverslips were embedded in Fluoromount-D with DAPI (Southern Biotech, Birmingham, AL, USA) on a microscopic glass slide. Then, the coverslips were analysed at 63x magnification with a Zeiss Axio Imager Z2 microscope (Zeiss, Sliedrecht, Netherlands) equipped with an ApoTome slider. The experiments were performed in triplicate. The statistical difference for the abnormal pattern was calculated for the wildtype compared to the TUBA4A-mutants using a two-way ANOVA followed by Tukey's post hoc test.

Supplementary table 2: **Quantification of microtubule morphology in COS1 cells overexpressed with TUBA4A-wildtype and TUBA4A variants.** Manual scoring of microtubule morphology was performed in COS-1 cells overexpressing wild-type TUBA4A or TUBA4A variants. Morphology ranged from normal, mild to abnormal cytoskeleton. The experiments were performed in triplicate. Raw counts for each scoring set are shown, together with the total number of cells scored per variant. Percentage values (pct_Normal, pct_Mild, pct_Abnormal) were calculated from the pooled counts and were used to generate the stacked bar plots shown in Figure 4.

Variant	Normal			Mild			Abnormal			Total cells counted	pct_Normal	pct_Mild	pct_Abnormal
TUBA4A wildtype	26	45	22	22	18	8	53	37	70	301	30.9	15.9	53.2
p.(Arg105Cys)	0	5	0	13	6	5	87	89	95	300	1.7	8.0	90.3
p.(Thr179Ile)	0	3	1	25	4	6	75	94	93	301	1.3	11.6	87.0
p.(Leu227Phe)	4	7	0	14	1	9	83	92	91	301	3.7	8.0	88.4
p.(Ser241Phe)	0	3	0	8	0	0	92	97	100	300	1.0	2.7	96.3
p.(Glu254Ala)	47	79	63	25	6	12	28	15	25	300	63.0	14.3	22.7
p.(Glu254Gly)	27	65	33	8	17	11	64	18	56	299	41.8	12.0	46.2
p.(Glu254Lys)	24	19	14	19	48	10	57	34	76	301	18.9	25.6	55.5
p.(Glu254Gln)	50	79	57	16	10	10	34	11	33	300	62.0	12.0	26.0
p.(Arg320Cys)	0	4	3	14	11	4	86	85	93	300	2.3	9.7	88.0
p.(Gly354Asp)	0	0	0	0	0	0	100	100	100	300	0.0	0.0	100.0

Western blotting confirmed comparable expression levels of all HA-tagged TUBA4A variants and wild-type TUBA4A in COS1 cells (image below), indicating that the observed morphological differences were not due to variable protein expression.

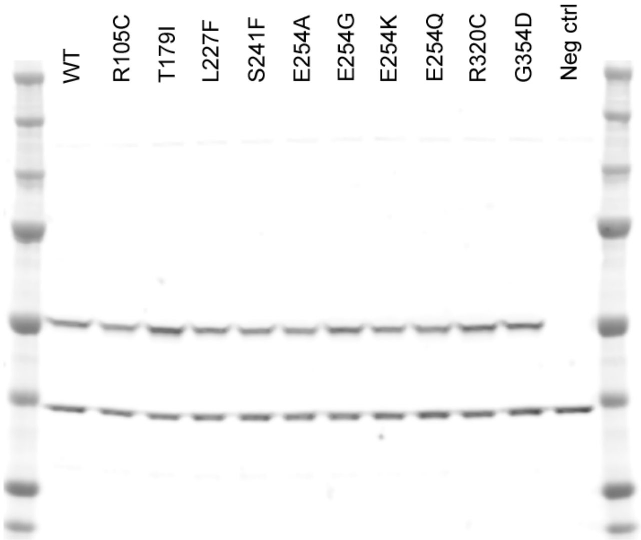

Supplementary Fig 4. Expression of HA-tagged TUBA4A variants in COS1 cells.

Western blot analysis of COS1 lysates transfected with wild-type and mutant HA-TUBA4A constructs. All variants showed comparable expression levels relative to wild type. The empty-vector control (Neg ctrl) confirms signal specificity. These results demonstrate that microtubule morphological abnormalities arise from variant-specific functional effects rather than differences in protein expression.

Correlation between genotype, *in silico* and *in vitro* observations

To explore any trend between variant location, predicted molecular impact and cellular phenotypes, we performed correlation analyses across three complementary datasets: (i) *in silico* predictions of protein destabilisation (FoldX ΔΔG; scaled z-scored per variant) and aggregation propensity (A4D), (ii) microtubule morphology scores from the *in vitro* overexpression assay, and (iii) TUBA4A domain location (GTPase, C-terminal, or GTP-binding). Morphology data were available only for 10 variants tested experimentally, and analyses involving morphology were therefore restricted to this subset. A composite morphology severity score (0–2) was derived from the proportional distribution of microtubule phenotypes (0 = normal, 1 = mild, 2 = abnormal), weighted by their respective percentages. Variants were categorised by domain using numeric codes (1 = GTPase, 2 = C-terminal, 3 = GTP-binding) to enable rank-based correlation testing. Because these variables included ordinal measures, proportions, and non-normally distributed computational scores, associations were assessed using Spearman's rank correlation. Partial correlations were computed using residuals from linear models regressing ΔΔG or aggregation propensity on domain, thereby estimating associations independent of domain location. All analyses were performed in R (v4.3.1) using pairwise complete observations.

Limitations: *In silico* correlations may be influenced by modelling assumptions, including reliance on alpha fold-derived structures, limited pLDDT coverage of some regions, and the simplification of domain categories. ΔΔG predictions model isolated monomers and do not incorporate chaperones or dimerisation partners, which may confound the relationship between variant location and predicted stability or aggregation scores.

Mechanistic correlations involving microtubule morphology are constrained by the limited number of variants tested (n=10), the semi-quantitative nature of the morphological scoring system, and potential batch effects inherent to overexpression assays. The distribution of variants across domains was uneven, with only two C-terminal variants available for morphology analysis, reducing the robustness of domain-based comparisons. One of these (p.Gly354Asp) was identified in our myotubulinopathy cohort, whereas the second (p.Arg320Cys) was included because of its established pathogenic association with ALS.

Code for analysis can be found at: https://github.com/RAVING-Informatics/TUBA4A

Supplementary table 3: Numerical domain classification where 1 = GTPase domain, 2 = C-terminal domain, 3 = GTP-binding region. Free energy change (scaled z-score): FoldX ΔΔG protein stability prediction expressed as a z-score. Higher values indicate a more destabilizing effect; negative values indicate a stabilizing or neutral effect. Aggregation propensity: Average Aggrescan4D aggregation score for each mutant protein. Microtubule morphology (EM): pct_normal: percentage of normal microtubules (0–100%). pct_mild: percentage of mildly abnormal microtubules. pct_abnormal: percentage of abnormal/severely altered microtubules. NA = variant not assessed in the EM assay.

Variant	Domain	Free energy change: scaled z score	Avg aggregation propensity for mutant protein	pct_normal microtubules	pct_mild microtubules	pct_abnormal microtubules
p.(Thr179Ile)	1	-0.532621545	-0.667710268	1.3	11.6	87
p.(Leu227Phe)	1	2.936511554	-0.647717411	3.7	8.0	88.4
p.(Ser241Phe)	1	-1.741054245	-0.650026563	1	2.7	96.3
p.(Glu254Lys)	1	-0.137162739	-0.695204911	18.9	25.6	55.5
p.(Glu254Gln)	1	-0.08307343	-0.687512277	62	12	26
p.(Glu254Gly)	1	0.562302776	-0.636965402	41.8	12	46.2
p.(Glu254Ala)	1	-0.092988309	-0.622576786	63	14.3	22.7
p.(His283Gln)	2	-0.4908509	-0.706720536	NA	NA	NA
p.(Glu284Lys)	2	-0.615806193	-0.627264063	NA	NA	NA
p.(Gly350Val)	2	0.735819445	-0.659463839	NA	NA	NA
p.(Gly354Asp)	2	-0.218812044	-0.653928571	0	0	100
p.(Gln11His)	3	0.122203393	-0.653240402	NA	NA	NA
p.(Ala12Thr)	3	0.266775252	-0.624107366	NA	NA	NA
p.(Arg105Cys)	1	0.84424862	-0.69006317	1.7	8	90.3
p.(Arg320Cys)	2	1.376456977	-0.693804688	2.3	9.7	88

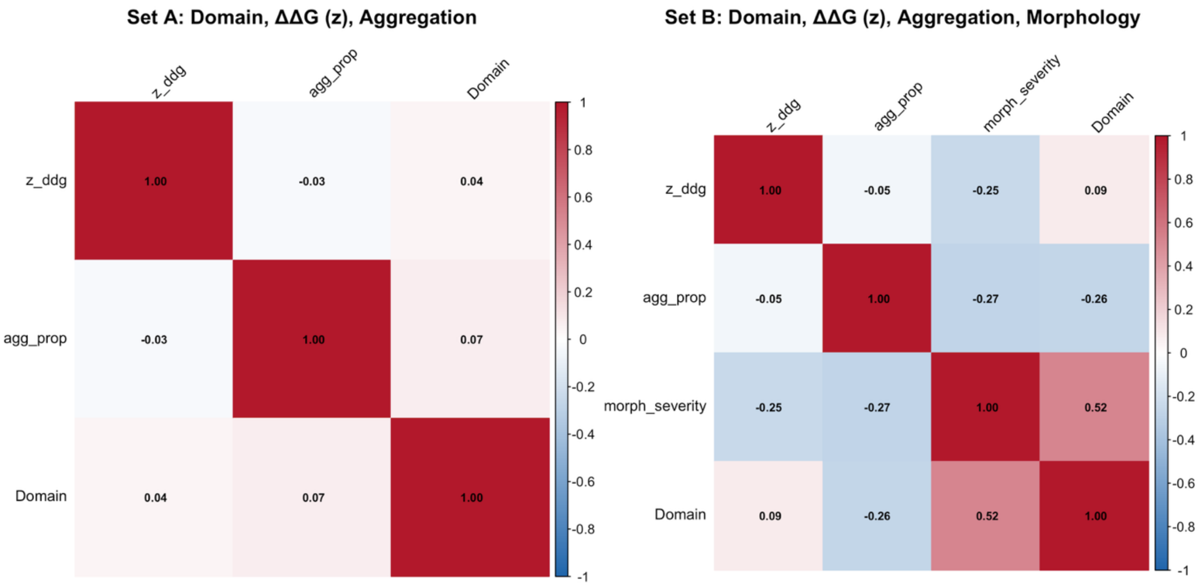

Supplementary Fig 5. Spearman correlation matrices summarising relationships between TUBA4A domain location, predicted protein stability (ΔΔG, z-scored), aggregation propensity, and microtubule morphology severity. Left (Set A): Analysis of all variants with complete in silico measurements (FoldX ΔΔG z-scores and Aggrescan4D aggregation scores) (n = 15; 13 myopathy variants, 2 ALS-associated variants). Domain location was encoded as an ordinal variable (1 = GTPase, 2 = C-terminal, 3 = GTP-binding). Colours represent Spearman ρ (–1 to +1). Only weak associations were observed between domain location and either ΔΔG or aggregation propensity in this set. Right (Set B): Analysis restricted to variants for which microtubule morphology data from COS-1 overexpression assays were available (n = 10; 8 myopathy variants, 2 ALS-associated variants). Morphology severity was calculated as a weighted composite score derived from the proportion of normal, mildly abnormal, and severely abnormal microtubules. In this subset, domain location showed a moderate positive correlation with morphology severity, whereas both ΔΔG and aggregation propensity showed weak negative correlations with the degree of microtubule disruption.

Quantification of microtubules on Electron Microscopy and analysis

Ultrathin resin sections from two patients (Pat.8 and Pat.9) and two age-matched controls were processed for transmission electron microscopy. Individual microtubules were identified in longitudinal and cross-sectional profiles, and their outer diameters were measured. Ten microtubules were identified in these two patients and only five in controls. To compare microtubule diameters between groups, we performed Welch's t-tests (unequal variances), reporting mean differences with 95% confidence intervals. Effect sizes were calculated as Cohen's *d*, and variance differences were evaluated using F-tests. Statistical analyses were conducted in R (v4.3.1) using the tidyverse, broom, and effsize packages. In longitudinal sections, microtubules were significantly thicker in patients than controls (mean 26.8 vs 23.1 nm; mean difference 3.79 nm, 95% CI 2.82–4.75; $p = 8.8 \times 10^{-8}$; Cohen's $d = 1.78$), with significantly greater variance ($p = 0.0486$). In cross-sections, the diameter increase was more modest but remained significant (mean 26.4 vs 24.9 nm; mean difference 1.53 nm, 95% CI 0.49–2.58; $p = 0.0055$; Cohen's $d = 0.78$). These quantitative findings confirm the presence of microtubule dilation and increased structural heterogeneity in patient muscle.

Supplementary table 4: Quantification data of microtubules.

Pat.9		Pat.8		Control.1		Control.2	
longitudinal (nm)	cross (nm)	longitudinal (nm)	cross (nm)	longitudinal (nm)	cross (nm)	longitudinal (nm)	cross (nm)
30.5389222	24.2284661	29.1110088	29.2952556	22.3859972	24.0442193	-	23.3532934
27.3146016	26.3473054	23.9981575	24.5048365	22.8926762	26.4394288		23.9981575
24.6430217	28.1437126	25.4260709	28.3740212	23.0769231	25.9327499		25
27.0382312	30.1243667	24.412713	28.0976509	21.2344542	25.0575772		

29.2031322	24.1363427	28.8346384		24.8733303	24.5969599		
24.4587748	27.6370336	25.241824		23.3532934	25.6563796		
22.2017504	22.0175035	25.4721327		23.6296638			
26.4394288	24.5508982	27.6370336		23.2151083			
29.2031322	29.8479963			24.5508982			
27.084293	25.9327499			21.3265776			
27.2685398	25.4721327						
26.8079226	25.0575772						
25.0575772	24.8733303						
24.9654537	20.8659604						
23.7678489	26.4854906						
26.3933671	26.0709351						
24.7351451	26.577614						
26.6697375	26.8539843						
28.1437126	26.3012437						
28.9267619	26.2551819						
26.6236757	26.6236757						
24.090281	29.5255643						
24.7351451	29.0188853						
26.577614	26.0709351						
22.9387379	26.9000461						
27.7752188	28.1437126						
29.1110088	25.0115154						
27.6830954							
25.7024413							
28.3279595							
27.084293							
24.5969599							
25.6563796							
24.9654537							
26.3933671							
26.0709351							
30.400737							
28.742515							
26.9000461							
21.6490097							
27.6830954							
27.3146016							
30.8152925							
27.0382312							
29.8479963							
26.8539843							

29.8479963							
33.8093045							
30.0322432							
26.0248733							
27.913404							
26.577614							
28.0976509							
26.8539843							
29.2491939							
29.3413174							
26.0248733							
26.0709351							
24.5969599							
24.0442193							

Code for analysis can be found at: https://github.com/RAVING-Informatics/TUBA4A

References

1. Pais LS, Snow H, Weisburd B, et al. seqr: A web-based analysis and collaboration tool for rare disease genomics. *Hum Mutat*. Jun 2022;43(6):698–707. doi:10.1002/humu.24366
2. Laurie S, Piscia D, Matalonga L, et al. The RD-Connect Genome-Phenome Analysis Platform: Accelerating diagnosis, research, and gene discovery for rare diseases. *Hum Mutat*. Jun 2022;43(6):717–733. doi:10.1002/humu.24353
3. Karczewski KJ, Francioli LC, Tiao G, et al. The mutational constraint spectrum quantified from variation in 141,456 humans. *Nature*. May 2020;581(7809):434–443. doi:10.1038/s41586-020-2308-7
4. Chen S, Francioli LC, Goodrich JK, et al. A genomic mutational constraint map using variation in 76,156 human genomes. *Nature*. Jan 2024;625(7993):92–100. doi:10.1038/s41586-023-06045-0
5. Kopanos C, Tsiolkas V, Kouris A, et al. VarSome: the human genomic variant search engine. *Bioinformatics*. Jun 1 2019;35(11):1978–1980. doi:10.1093/bioinformatics/bty897
6. Delgado J, Radusky LG, Cianferoni D, Serrano L. FoldX 5.0: working with RNA, small molecules and a new graphical interface. *Bioinformatics*. Oct 15 2019;35(20):4168–4169. doi:10.1093/bioinformatics/btz184
7. Akdel M, Pires DEV, Pardo EP, et al. A structural biology community assessment of AlphaFold2 applications. *Nat Struct Mol Biol*. Nov 2022;29(11):1056–1067. doi:10.1038/s41594-022-00849-w
8. Ravenscroft G, Miyatake S, Lehtokari VL, et al. Mutations in KLHL40 are a frequent cause of severe autosomal-recessive nemaline myopathy. *Am J Hum Genet*. Jul 11 2013;93(1):6–18. doi:10.1016/j.ajhg.2013.05.004
9. Mao K, Borel C, Ansar M, et al. FOXI3 pathogenic variants cause one form of craniofacial microsomia. *Nat Commun*. Apr 11 2023;14(1):2026. doi:10.1038/s41467-023-37703-6
10. Badaczewska-Dawid AE, Kuriata A, Pintado-Grima C, et al. A3D Model Organism Database (A3D-MODB): a database for proteome aggregation predictions in model organisms. *Nucleic Acids Res*. Jan 5 2024;52(D1):D360–D367. doi:10.1093/nar/gkad942
11. Kuriata A, Iglesias V, Pujols J, Kurcinski M, Kmiecik S, Ventura S. Aggrescan3D (A3D) 2.0: prediction and engineering of protein solubility. *Nucleic Acids Res*. Jul 2 2019;47(W1):W300–W307. doi:10.1093/nar/gkz321
12. Zalewski M, Iglesias V, Barcenas O, Ventura S, Kmiecik S. Aggrescan4D: A comprehensive tool for pH-dependent analysis and engineering of protein aggregation propensity. *Protein Sci*. Oct 2024;33(10):e5180. doi:10.1002/pro.5180
13. Jumper J, Evans R, Pritzel A, et al. Highly accurate protein structure prediction with AlphaFold. *Nature*. Aug 2021;596(7873):583–589. doi:10.1038/s41586-021-03819-2

14. Diao L, Zheng W, Zhao Q, et al. Cryo-EM of α-tubulin isotype-containing microtubules revealed a contracted structure of α4A/β2A microtubules. *Acta Biochim Biophys Sin (Shanghai)*. Oct 25 2023;55(10):1551–1560. doi:10.3724/abbs.2023130
15. Sehnal D, Bittrich S, Deshpande M, et al. Mol* Viewer: modern web app for 3D visualization and analysis of large biomolecular structures. *Nucleic Acids Res*. Jul 2 2021;49(W1):W431–w437. doi:10.1093/nar/gkab314
16. Smith BN, Ticozzi N, Fallini C, et al. Exome-wide rare variant analysis identifies TUBA4A mutations associated with familial ALS. *Neuron*. Oct 22 2014;84(2):324–31. doi:10.1016/j.neuron.2014.09.027
