# Supplementary material for: Missense variants in TUBA4A cause myo-tubulinopathies
Source: Brain. Author manuscript; Available in PMC 2026 Aug 29. (PMC13524141; doi:10.1093/brain/awag059)
Supplement: Supplementary file 3 [file NIHMS2203371-supplement-Supplementary_Table.pdf]

**Supplementary Table: Assessment of variants in *TUBA4A* observed in our cohort. AL=Acceptor Loss, AG=Acceptor Gain, AR = Autosomal recessive, AD = Autosomal dominant, n=number of confirmed affected individuals, N=number of families, NA=Not assessed.**

| | | Population fequency | Raw Classification | | *In silico* evidence | | | | | | | | *In vitro* evidence | | Clinical evidence | | | Variants in other alphaTubulins | Revised Classification | |
|---|---|---|---|---|---|---|---|---|---|---|---|---|---|---|---|---|---|---|---|---|
| **TUBA4A variants NM_006000.3** | **hg38 coordinates** | **gnomAD4.1.0** | **Assertion** | **Rules** | **AlphaMissense** | **CADD** | **REVEL** | **spliceAI** | **Free Energy** | **Polar contacts** | **Steric hindrance** | **Aggrescan 4D of residue** | **Morphology in COS1** | **Proband IDs** | **Phenotype** | **type/segregation** | **n[N]** | | **Assertion** | **add Rules** |
| ***de novo*** | | | | | | | | | | | | | | | | | | | | |
| c.33G>C(p.Gln11His) | chr2-219252201-C-G | 0 | VUS | PP3_Moderate,PM2_Supporting,PP2_Supporting | 0.9967 | 24.4 | 0.808 | 0 | Positive | addition | Yes | "-0.742" (Soluble residue) | NA | Pat.24 | Multisystem proteinopathy | heterozygous | 1[1] | | LP | PS2_strong |
| c.679C>T(p.Leu227Phe)* | chr2-219251020-G-A | 0 | VUS | PP3_Moderate,PM2_Supporting,PP2_Supporting | 0.9921 | 26.7 | 0.755 | 0 | High Positive | no change | Yes | 0.5505 (aggregation prone residue) | abnormal-mild | Pat.7 | Myopathy | heterozygous | 1[1] | *TUBA1A* :(p.(Leu227Gln)) is a VUS | P | PS2_strong, PS3_strong, PP1_supporting |
| c.761A>G(p.Glu254Gly) | chr2-219250938-T-C | 0 | LP | PP3_Strong,PM2_Supporting,PP2_Supporting | 0.989 | 29.3 | 0.929 | AL=0.02 | Positive | loss | No | "-0.5888" (Soluble residue) | normal-mild | Pat.10 | Myopathy | heterozygous | 1[1] | | P | PS2_strong, PS3_strong |
| c.760G>A(p.Glu254Lys) | chr2-219250939-C-T | 0 | VUS | PP3_Moderate,PM2_Supporting,PP2_Supporting | 0.999 | 26.7 | 0.846 | 0 | none | loss | No | "-1.4057" (Soluble residue) | mild | Pat.8 | Myopathy | heterozygous | 1[1] | | P | PS2_strong, PS3_strong |
| c.1049G>T(p.Gly350Val) | chr2-219250650-C-A | 0 | LP | PP3_Strong,PM2_Supporting,PP2_Supporting | 0.999 | 26 | 0.893 | 0 | Positive | no change | Yes | "-0.5039" (soluble residue) | NA | Pat.25 | Myopathy | heterozygous | 1[1] | *TUBA1A* :(p.(Gly350Val)) is pathogenic | P | PS2_strong |
| ***Sporadic*** | | | | | | | | | | | | | | | | | | | | |
| c.536C>T(p.Thr179Ile)* | chr2-219251163-G-A | 0 | VUS | PP3_Moderate,PM2_Supporting,PP2_Supporting | 0.9657 | 25.3 | 0.731 | AL=0.06/AG=0.02 | Negative | none | No | 2.1503 (aggregation prone residue) | abnormal-mild | Pat.5 | Myopathy | heterozygous | 1[1] | | P | PS3_strong, PP1_supporting |
| c.679C>T(p.Leu227Phe)* | chr2-219251020-G-A | 0 | VUS | PP3_Moderate,PM2_Supporting,PP2_Supporting | 0.9921 | 26.7 | 0.755 | 0 | High Positive | no change | Yes | 0.5505 (aggregation prone residue) | abnormal-mild | Pat.6 | Myopathy | heterozygous | 1[1] | *TUBA1A* :(p.(Leu227Gln)) is a VUS | P | PS2_strong, PS3_strong, PP1_supporting |
| c.761A>C(p.Glu254Ala) | chr2-219250938-T-G | 0 | LP | PP3_Strong,PM2_Supporting,PP2_Supporting | 0.9895 | 27.4 | 0.941 | 0 | none | loss | No | "-0.6042" (Soluble residue) | normal-mild | Pat.11 | Myopathy | heterozygous | 1[1] | | P | PS3_strong |
| c.760G>C(p.Glu254Gln) | chr2-219250939-C-G | 0 | VUS | PP3_Moderate,PM2_Supporting,PP2_Supporting | 0.9905 | 23.5 | 0.729 | 0 | none | loss | No | "-1.612" (Soluble residue) | normal-mild | Pat.9 | Myopathy | heterozygous | 1[1] | | LP | PS3_strong |
| c.849C>G(p.His283Gln) | chr2-219250850-G-C | 0 | LP | PP3_Strong,PM2_Supporting,PP2_Supporting | 0.9923 | 17.92 | 0.735 | 0 | Negative | addition | No | "-1.662" (Soluble residue) | NA | Pat.12 | Myopathy | heterozygous | 1[1] | *TUBA1A:* (p.(His283Arg)) is Liklely pathogenic | LP | none |
| c.850G>A(p.Glu284Lys)* | chr2-219250849-C-T | 0 | VUS | PP3_Moderate,PM2_Moderate,PP2_Supporting | 0.9894 | 27.1 | 0.824 | 0 | Negative | none | No | "-2.6099" (Soluble residue) | NA | Pat.13 and Pat.14 | Myopathy | heterozygous | 2[2] | *TUBA8* :(p.(Glu284Lys)) is VUS | LP | PP1_supporting |
| ***Autosomal Recessive*** | | | | | | | | | | | | | | | | | | | | |
| c.34G>A(p.Ala12Thr) | chr2-219252200-C-T | 0 | VUS | PP3_Moderate,PM2_Supporting,PP2_Supporting | 0.9451 | 26.8 | 0.832 | 0 | Positive | addition | Yes | "-0.4499" (Soluble residue) | NA | Pat.26 | Multisystem proteinopathy | homozygous/AR | 1[1] | | VUS | none |
| c.722C>T(p.Ser241Phe) | chr2-219250977-G-A | 0 | VUS | PP3_Moderate,PM2_Supporting,PP2_Supporting | 0.9989 | 28.3 | 0.795 | 0 | High Negative | loss | Yes | 0 (Soluble residue) | abnormal | Pat.21 | Myopathy | homozygous/AR | 1[1] | | P | PS3_strong, change PP3_strong |
| c.1061G>A(p.Gly354Asp) | chr2-219250638-C-T | 0 | LP | PP3_Strong,PM2_Supporting,PP2_Supporting | 0.9996 | 26.1 | 0.901 | 0 | Negative | addition | Yes | 0 (Soluble residue) | abnormal | Pat.22 and Pat.23 | Myopathy | homozygous/AR | 2[1] | | P | PS3_strong |
| ***Autosomal Dominant*** | | | | | | | | | | | | | | | | | | | | |
| c.536C>T(p.Thr179Ile)* | chr2-219251163-G-A | 0 | VUS | PP3_Moderate,PM2_Supporting,PP2_Supporting | 0.9657 | 25.3 | 0.731 | AL=0.06/AG=0.02 | Negative | none | No | 2.1503 (aggregation prone residue) | abnormal-mild | Pat.2 | Myopathy | heterozygous/AD | 4[1] | | P | PS3_strong, PP1_supporting |
| c.850G>A(p.Glu284Lys)* | chr2-219250849-C-T | 0 | VUS | PP3_Moderate,PM2_Supporting,PP2_Supporting | 0.9894 | 27.1 | 0.824 | 0 | Negative | none | No | "-2.6099" (Soluble residue) | NA | Pat.15, Pat16A, Pat.16B, Pat.17 and Pat.18 | Myopathy | heterozygous/AD | 11[3] | *TUBA8* :(p.(Glu284Lys)) is VUS | LP | PP1_supporting |

* indicates recurring variants
